# Supplementary material for: Cognitive Function Is Associated with the Genetically Determined Efficiency of DNA Repair Mechanisms
Source: Genes (Basel). 2024 Jan 24;15(2):153. doi: 10.3390/genes15020153 (PMC10888195; doi:10.3390/genes15020153)

Table S1. Prevalence of SNPs considered for inclusion in analyses. Of those rs77542170, rs200844166, rs200495564, and rs121908381 were excluded due to low prevalence (n<50).

	Female (N=264,576)	Male (N=223,437)	Total	p value
**rs1052133**				0.662
0	155833 (59.0%)	131338 (58.9%)	287171 (58.9%)	
1	93856 (35.5%)	79527 (35.6%)	173383 (35.6%)	
2	14534 (5.5%)	12237 (5.5%)	26771 (5.5%)	
N-Miss	353	335	688	
**rs104893751**				0.166
0	262192 (99.2%)	221368 (99.2%)	483560 (99.2%)	
1	2129 (0.8%)	1851 (0.8%)	3980 (0.8%)	
2	2 (0.0%)	6 (0.0%)	8 (0.0%)	
N-Miss	253	212	465	
**rs7402844**				0.162
0	18177 (6.9%)	15398 (6.9%)	33575 (6.9%)	
1	100024 (37.9%)	83880 (37.6%)	183904 (37.7%)	
2	146048 (55.3%)	123885 (55.5%)	269933 (55.4%)	
N-Miss	327	274	601	
**rs5745906**				0.438
0	263993 (99.8%)	222971 (99.8%)	486964 (99.8%)	
1	537 (0.2%)	434 (0.2%)	971 (0.2%)	
2	0 (0.0%)	1 (0.0%)	1 (0.0%)	
N-Miss	46	31	77	
**rs6601606**				0.008
0	256187 (96.9%)	216671 (97.0%)	472858 (96.9%)	
1	8073 (3.1%)	6547 (2.9%)	14620 (3.0%)	
2	167 (0.1%)	112 (0.1%)	279 (0.1%)	
N-Miss	149	107	256	
**rs10013040**				0.013
0	159465 (60.3%)	133740 (59.9%)	293205 (60.1%)	
1	91510 (34.6%)	78039 (35.0%)	169549 (34.8%)	
2	13333 (5.0%)	11419 (5.1%)	24752 (5.1%)	
N-Miss	268	239	507	
**rs13112390**				0.033

	Female (N=264,576)	Male (N=223,437)	Total	p value
0	12130 (4.6%)	10104 (4.5%)	22234 (4.6%)	
1	88087 (33.5%)	73697 (33.2%)	161784 (33.3%)	
2	162942 (61.9%)	138364 (62.3%)	301306 (62.1%)	
N-Miss	1417	1272	2689	
**rs13112358**				0.226
0	16042 (6.1%)	13423 (6.0%)	29465 (6.1%)	
1	96709 (36.7%)	81241 (36.5%)	177950 (36.6%)	
2	151020 (57.3%)	128052 (57.5%)	279072 (57.4%)	
N-Miss	805	721	1526	
**rs1395479**				0.324
0	145447 (55.1%)	122357 (54.9%)	267804 (55.0%)	
1	100645 (38.1%)	85374 (38.3%)	186019 (38.2%)	
2	17948 (6.8%)	15256 (6.8%)	33204 (6.8%)	
N-Miss	536	450	986	
**rs34612342**				0.494
0	263195 (99.6%)	222251 (99.6%)	485446 (99.6%)	
1	1131 (0.4%)	984 (0.4%)	2115 (0.4%)	
N-Miss	250	202	452	
**rs200165598**				0.559
0	264356 (99.9%)	223258 (99.9%)	487614 (99.9%)	
1	193 (0.1%)	153 (0.1%)	346 (0.1%)	
N-Miss	27	26	53	
**rs77542170**				0.840
0	24929 (100.0%)	24913 (99.9%)	49842 (99.9%)	
1	12 (0.0%)	13 (0.1%)	25 (0.1%)	
N-Miss	239635	198511	438146	
**rs200844166**				0.068
0	239552 (100.0%)	198436 (100.0%)	437988 (100.0%)	
1	3 (0.0%)	8 (0.0%)	11 (0.0%)	
N-Miss	25021	24993	50014	
**rs200495564**				0.401
0	264327 (100.0%)	223195 (100.0%)	487522 (100.0%)	

	**Female (N=264,576)**	**Male (N=223,437)**	**Total**	**p value**
1	7 (0.0%)	9 (0.0%)	16 (0.0%)	
N-Miss	242	233	475	
**rs121908381**				1.000
0	24946 (100.0%)	24939 (100.0%)	49885 (100.0%)	
1	1 (0.0%)	1 (0.0%)	2 (0.0%)	
N-Miss	239629	198497	438126	
**rs150766139**				0.496
0	263695 (99.7%)	222674 (99.7%)	486369 (99.7%)	
1	827 (0.3%)	723 (0.3%)	1550 (0.3%)	
N-Miss	54	40	94	
**rs2516739**				0.045
0	160213 (60.6%)	135929 (60.9%)	296142 (60.8%)	
1	90352 (34.2%)	75917 (34.0%)	166269 (34.1%)	
2	13676 (5.2%)	11275 (5.1%)	24951 (5.1%)	
N-Miss	335	316	651	

Table S2. Ethnic background of included participants.

	**Female (N=264,576)**	**Male (N=223,437)**	**Total (N=488,013)**
**Ethnicity**			
African	1554 (0.6%)	1649 (0.7%)	3203 (0.7%)
Any other Asian background	793 (0.3%)	953 (0.4%)	1746 (0.4%)
Any other Black background	78 (0.0%)	40 (0.0%)	118 (0.0%)
Any other mixed background	624 (0.2%)	368 (0.2%)	992 (0.2%)
Any other white background	9671 (3.7%)	6133 (2.7%)	15804 (3.2%)
Asian or Asian British	23 (0.0%)	19 (0.0%)	42 (0.0%)
Bangladeshi	67 (0.0%)	154 (0.1%)	221 (0.0%)
Black or Black British	17 (0.0%)	9 (0.0%)	26 (0.0%)
British	233099 (88.2%)	197712 (88.6%)	430811 (88.4%)
Caribbean	2708 (1.0%)	1587 (0.7%)	4295 (0.9%)
Chinese	939 (0.4%)	563 (0.3%)	1502 (0.3%)
Do not know	103 (0.0%)	101 (0.0%)	204 (0.0%)
Indian	2801 (1.1%)	2914 (1.3%)	5715 (1.2%)
Irish	6625 (2.5%)	6119 (2.7%)	12744 (2.6%)
Mixed	28 (0.0%)	18 (0.0%)	46 (0.0%)
Other ethnic group	2471 (0.9%)	1881 (0.8%)	4352 (0.9%)
Pakistani	673 (0.3%)	1073 (0.5%)	1746 (0.4%)
Prefer not to answer	673 (0.3%)	908 (0.4%)	1581 (0.3%)
White	233 (0.1%)	311 (0.1%)	544 (0.1%)
White and Asian	462 (0.2%)	339 (0.2%)	801 (0.2%)
White and Black African	279 (0.1%)	123 (0.1%)	402 (0.1%)
White and Black Caribbean	375 (0.1%)	221 (0.1%)	596 (0.1%)
N-Miss	280	242	522

Table S3. Univariate associations between rs1052133 (*OGG1*) and cognitive outcomes after controlling for age (centered on 60 years), sex, and education. Statistics reported include unstandardized Beta estimates and p values. Asterisks indicate the significance levels at *p<0.05; **p<0.0035 (Bonferroni corrected); ***p<0.00001.

	Cognitive Measures				
SNP variants (1/2 alleles vs none)	FIQ	SDMT	MATCH	TRAIL1	TRAIL2
age_60	-0.017^{***}	-0.271^{***}	3.625^{***}	0.494^{***}	1.031^{***}
	p = 0.000	p = 0.000	p = 0.000	p = 0.000	p = 0.000
sexMale	0.190^{***}	0.028	-18.002^{***}	-2.257^{***}	-2.488^{***}
	p = 0.000	p = 0.317	p = 0.000	p = 0.000	p = 0.000
educationSecondary	1.292^{***}	2.073^{***}	-25.243^{***}	-4.513^{***}	-13.224^{***}
	p = 0.000	p = 0.000	p = 0.000	p = 0.000	p = 0.000
educationProf cert/dip	0.909^{***}	1.308^{***}	-18.398^{***}	-3.129^{***}	-8.682^{***}
	p = 0.000	p = 0.000	p = 0.000	p = 0.000	p = 0.000
educationTertiary	2.241^{***}	2.924^{***}	-32.128^{***}	-6.352^{***}	-18.216^{***}
	p = 0.000	p = 0.000	p = 0.000	p = 0.000	p = 0.000
educationUnknown	0.866^{***}	0.813^{**}	11.424^{***}	-3.556^{***}	-7.963^{***}
	p = 0.000	p = 0.00002	p = 0.000	p = 0.00000	p = 0.000
rs10521331	-0.025^{*}	-0.056	0.650	0.082	0.066
	p = 0.031	p = 0.056	p = 0.058	p = 0.388	p = 0.676
rs10521332	-0.019	0.012	2.331^{**}	0.253	0.276
	p = 0.429	p = 0.846	p = 0.002	p = 0.203	p = 0.403
Constant	3.708^{***}	16.400^{***}	601.641^{***}	47.138^{***}	86.341^{***}
	p = 0.000	p = 0.000	p = 0.000	p = 0.000	p = 0.000
Observations	121,005	115,955	482,145	101,857	101,855
Log Likelihood	-249,958.100	-342,928.900	-2,959,828.000	-415,567.100	-466,943.100
Akaike Inf. Crit.	499,934.200	685,875.800	5,919,673.000	831,152.200	933,904.300

Note: *p<0.05;* ***p<0.0035;*** p<1e-05

Table S4. Univariate association between rs104893751 (*OGG1*) and cognitive outcomes after controlling for age (centered on 60 years), sex, and education. Statistics reported include unstandardized Beta estimates and p values. Asterisks indicate the significance levels at *p<0.05; **p<0.0035 (Bonferroni corrected); ***p<0.00001.

	Cognitive Measures				
SNP variants (1/2 alleles vs none)	FIQ	SDMT	MATCH	TRAIL1	TRAIL2
age_60	-0.017***	-0.271***	3.624***	0.493***	1.030***
	p = 0.000	p = 0.000	p = 0.000	p = 0.000	p = 0.000
sexMale	0.189***	0.027	-17.992***	-2.254***	-2.479***
	p = 0.000	p = 0.324	p = 0.000	p = 0.000	p = 0.000
educationSecondary	1.293***	2.070***	-25.210***	-4.511***	-13.219***
	p = 0.000	p = 0.000	p = 0.000	p = 0.000	p = 0.000
educationProf cert/dip	0.910***	1.307***	-18.380***	-3.121***	-8.668***
	p = 0.000	p = 0.000	p = 0.000	p = 0.000	p = 0.000
educationTertiary	2.241***	2.922***	-32.089***	-6.342***	-18.203***
	p = 0.000	p = 0.000	p = 0.000	p = 0.000	p = 0.000
educationUnknown	0.865***	0.820**	11.539***	-3.555***	-7.918***
	p = 0.000	p = 0.00002	p = 0.000	p = 0.00000	p = 0.000
rs1048937511	0.031	-0.009	-5.855**	0.263	-0.069
	p = 0.606	p = 0.953	p = 0.002	p = 0.591	p = 0.933
rs1048937512	1.359	-1.418	-51.562	-3.458	-9.546
	p = 0.082	p = 0.456	p = 0.194	p = 0.629	p = 0.421
Constant	3.698***	16.383***	602.016***	47.167***	86.358***
	p = 0.000	p = 0.000	p = 0.000	p = 0.000	p = 0.000
Observations	121,060	116,004	482,371	101,898	101,896
Log Likelihood	-250,073.700	-343,068.400	-2,961,223.000	-415,720.100	-467,107.300
Akaike Inf. Crit.	500,165.400	686,154.700	5,922,464.000	831,458.100	934,232.700

Note: *p<0.05;* ***p<0.0035;*** p<1e-05

Table S5. Univariate association between rs7402844 (*NEIL1*) and cognitive outcomes after controlling for age (centered on 60 years), sex, and education. Statistics reported include unstandardized Beta estimates and p values. Asterisks indicate the significance levels at *p<0.05; **p<0.0035 (Bonferroni corrected); ***p<0.00001.

	Cognitive Measures				
SNP variants (1/2 alleles vs none)	FIQ	SDMT	MATCH	TRAIL1	TRAIL2
age_60	-0.017***	-0.271***	3.631***	0.493***	1.030***
	p = 0.000	p = 0.000	p = 0.000	p = 0.000	p = 0.000
sexMale	0.189***	0.026	-17.987***	-2.257***	-2.487***
	p = 0.000	p = 0.355	p = 0.000	p = 0.000	p = 0.000
educationSecondary	1.293***	2.073***	-25.203***	-4.516***	-13.238***
	p = 0.000	p = 0.000	p = 0.000	p = 0.000	p = 0.000
educationProf cert/dip	0.910***	1.313***	-18.419***	-3.140***	-8.692***
	p = 0.000	p = 0.000	p = 0.000	p = 0.000	p = 0.000
educationTertiary	2.242***	2.924***	-32.165***	-6.352***	-18.228***
	p = 0.000	p = 0.000	p = 0.000	p = 0.000	p = 0.000
educationUnknown	0.868***	0.814**	11.206***	-3.556***	-7.977***
	p = 0.000	p = 0.00002	p = 0.000	p = 0.00000	p = 0.000
rs74028441	0.083**	0.009	-9.041***	-0.131	-0.665*
	p = 0.0004	p = 0.879	p = 0.000	p = 0.486	p = 0.033
rs74028442	0.105***	0.055	-11.351***	-0.154	-0.923**
	p = 0.00001	p = 0.324	p = 0.000	p = 0.402	p = 0.003
Constant	3.608***	16.348***	611.718***	47.313***	87.147***
	p = 0.000	p = 0.000	p = 0.000	p = 0.000	p = 0.000
Observations	121,035	115,982	482,238	101,885	101,883
Log Likelihood	-250,006.300	-342,998.500	-2,960,249.000	-415,679.200	-467,031.900
Akaike Inf. Crit.	500,030.600	686,015.100	5,920,517.000	831,376.400	934,081.800

Note: *p<0.05;* ***p<0.0035;*** p<1e-05

Table S6. Univariate association between rs5745906 (*NEIL1*) and cognitive outcomes after controlling for age (centered on 60 years), sex, and education. Statistics reported include unstandardized Beta estimates and p values. Asterisks indicate the significance levels at *p<0.05; **p<0.0035 (Bonferroni corrected); ***p<0.00001.

	Cognitive Measures				
SNP variants (1/2 alleles vs none)	FIQ	SDMT	MATCH	TRAIL1	TRAIL2
age_60	-0.017***	-0.271***	3.625***	0.493***	1.030***
	p = 0.000	p = 0.000	p = 0.000	p = 0.000	p = 0.000
sexMale	0.190***	0.026	-18.005***	-2.253***	-2.489***
	p = 0.000	p = 0.339	p = 0.000	p = 0.000	p = 0.000
educationSecondary	1.292***	2.072***	-25.208***	-4.512***	-13.232***
	p = 0.000	p = 0.000	p = 0.000	p = 0.000	p = 0.000
educationProf cert/dip	0.910***	1.309***	-18.366***	-3.127***	-8.676***
	p = 0.000	p = 0.000	p = 0.000	p = 0.000	p = 0.000
educationTertiary	2.241***	2.922***	-32.094***	-6.348***	-18.215***
	p = 0.000	p = 0.000	p = 0.000	p = 0.000	p = 0.000
educationUnknown	0.863***	0.807**	11.535***	-3.525***	-7.924***
	p = 0.000	p = 0.00002	p = 0.000	p = 0.00000	p = 0.000
rs57459061	-0.001	0.268	-6.352	-0.114	-1.597
	p = 0.991	p = 0.368	p = 0.080	p = 0.907	p = 0.321
rs57459062			156.615		
			p = 0.163		
Constant	3.698***	16.382***	601.985***	47.174***	86.379***
	p = 0.000	p = 0.000	p = 0.000	p = 0.000	p = 0.000
Observations	121,160	116,102	482,754	101,986	101,984
Log Likelihood	-250,276.100	-343,353.700	-2,963,533.000	-416,087.900	-467,517.300
Akaike Inf. Crit.	500,568.200	686,723.400	5,927,084.000	832,191.700	935,050.600
Note:					*p<0.05;* ***p<0.0035;*** p<1e-05

Table S7. Univariate association between rs6601606 (*NEIL2*) and cognitive outcomes after controlling for age (centered on 60 years), sex, and education. Statistics reported include unstandardized Beta estimates and p values. Asterisks indicate the significance levels at *p<0.05; **p<0.0035 (Bonferroni corrected); ***p<0.00001.

	Cognitive Measures				
SNP variants (1/2 alleles vs none)	FIQ	SDMT	MATCH	TRAIL1	TRAIL2
age_60	-0.017^{***}	-0.271^{***}	3.627^{***}	0.493^{***}	1.030^{***}
	p = 0.000	p = 0.000	p = 0.000	p = 0.000	p = 0.000
sexMale	0.190^{***}	0.026	-17.977^{***}	-2.254^{***}	-2.482^{***}
	p = 0.000	p = 0.341	p = 0.000	p = 0.000	p = 0.000
educationSecondary	1.292^{***}	2.072^{***}	-25.210^{***}	-4.510^{***}	-13.226^{***}
	p = 0.000	p = 0.000	p = 0.000	p = 0.000	p = 0.000
educationProf cert/dip	0.911^{***}	1.310^{***}	-18.394^{***}	-3.138^{***}	-8.691^{***}
	p = 0.000	p = 0.000	p = 0.000	p = 0.000	p = 0.000
educationTertiary	2.241^{***}	2.923^{***}	-32.112^{***}	-6.348^{***}	-18.215^{***}
	p = 0.000	p = 0.000	p = 0.000	p = 0.000	p = 0.000
educationUnknown	0.869^{***}	0.814^{**}	11.390^{***}	-3.555^{***}	-7.962^{***}
	p = 0.000	p = 0.00002	p = 0.000	p = 0.00000	p = 0.000
rs66016061	-0.192^{***}	-0.021	7.526^{***}	-0.037	0.455
	p = 0.000	p = 0.800	p = 0.000	p = 0.892	p = 0.317
rs66016062	-1.590^{***}	-0.656	26.378^{**}	2.448	0.576
	p = 0.000	p = 0.301	p = 0.0002	p = 0.257	p = 0.872
Constant	3.703^{***}	16.383^{***}	601.738^{***}	47.175^{***}	86.359^{***}
	p = 0.000	p = 0.000	p = 0.000	p = 0.000	p = 0.000
Observations	121,115	116,059	482,573	101,946	101,944
Log Likelihood	-250,158.000	-343,223.600	-2,962,352.000	-415,916.500	-467,329.300
Akaike Inf. Crit.	500,333.900	686,465.300	5,924,722.000	831,851.100	934,676.500

Note: *p<0.05;* ***p<0.0035;*** p<1e-05

Table S8. Univariate association between rs10013040 (*NEIL3*) and cognitive outcomes after controlling for age (centered on 60 years), sex, and education. Statistics reported include unstandardized Beta estimates and p values. Asterisks indicate the significance levels at *p<0.05; **p<0.0035 (Bonferroni corrected); ***p<0.00001.

	Cognitive Measures				
SNP variants (1/2 alleles vs none)	FIQ	SDMT	MATCH	TRAIL1	TRAIL2
age_60	-0.017***	-0.271***	3.625***	0.493***	1.030***
	p = 0.000	p = 0.000	p = 0.000	p = 0.000	p = 0.000
sexMale	0.189***	0.026	-17.981***	-2.254***	-2.483***
	p = 0.000	p = 0.344	p = 0.000	p = 0.000	p = 0.000
educationSecondary	1.293***	2.072***	-25.232***	-4.516***	-13.248***
	p = 0.000	p = 0.000	p = 0.000	p = 0.000	p = 0.000
educationProf cert/dip	0.910***	1.312***	-18.390***	-3.134***	-8.691***
	p = 0.000	p = 0.000	p = 0.000	p = 0.000	p = 0.000
educationTertiary	2.242***	2.923***	-32.104***	-6.347***	-18.228***
	p = 0.000	p = 0.000	p = 0.000	p = 0.000	p = 0.000
educationUnknown	0.867***	0.814**	11.499***	-3.551***	-7.973***
	p = 0.000	p = 0.00002	p = 0.000	p = 0.00000	p = 0.000
rs100130401	0.020	0.082*	-0.950*	-0.133	-0.248
	p = 0.087	p = 0.006	p = 0.006	p = 0.165	p = 0.117
rs100130402	-0.013	0.076	-1.118	0.211	0.156
	p = 0.612	p = 0.228	p = 0.135	p = 0.308	p = 0.650
Constant	3.691***	16.349***	602.374***	47.211***	86.465***
	p = 0.000	p = 0.000	p = 0.000	p = 0.000	p = 0.000
Observations	121,065	116,012	482,332	101,904	101,902
Log Likelihood	-250,084.900	-343,093.000	-2,960,970.000	-415,757.500	-467,144.700
Akaike Inf. Crit.	500,187.800	686,204.000	5,921,959.000	831,533.000	934,307.500
Note:					*p<0.05;* ***p<0.0035;*** p<1e-05

Table S9. Univariate association between rs13112390 (*NEIL3*) and cognitive outcomes after controlling for age (centered on 60 years), sex, and education. Statistics reported include unstandardized Beta estimates and p values. Asterisks indicate the significance levels at *p<0.05; **p<0.0035 (Bonferroni corrected); ***p<0.00001.

	Cognitive Measures				
SNP variants (1/2 alleles vs none)	FIQ	SDMT	MATCH	TRAIL1	TRAIL2
age_60	-0.017***	-0.271***	3.627***	0.494***	1.030***
	p = 0.000	p = 0.000	p = 0.000	p = 0.000	p = 0.000
sexMale	0.190***	0.026	-17.979***	-2.250***	-2.487***
	p = 0.000	p = 0.356	p = 0.000	p = 0.000	p = 0.000
educationSecondary	1.294***	2.072***	-25.175***	-4.508***	-13.182***
	p = 0.000	p = 0.000	p = 0.000	p = 0.000	p = 0.000
educationProf cert/dip	0.910***	1.309***	-18.338***	-3.164***	-8.656***
	p = 0.000	p = 0.000	p = 0.000	p = 0.000	p = 0.000
educationTertiary	2.243***	2.921***	-32.094***	-6.347***	-18.173***
	p = 0.000	p = 0.000	p = 0.000	p = 0.000	p = 0.000
educationUnknown	0.875***	0.814**	11.533***	-3.552***	-7.924***
	p = 0.000	p = 0.00002	p = 0.000	p = 0.00000	p = 0.000
rs131123901	0.088**	0.057	-3.378**	-0.039	-0.298
	p = 0.002	p = 0.410	p = 0.00003	p = 0.865	p = 0.427
rs131123902	0.102**	0.081	-4.790***	-0.029	-0.503
	p = 0.0002	p = 0.230	p = 0.000	p = 0.895	p = 0.168
Constant	3.603***	16.313***	606.050***	47.204***	86.748***
	p = 0.000	p = 0.000	p = 0.000	p = 0.000	p = 0.000
Observations	120,515	115,488	480,173	101,436	101,434
Log Likelihood	-248,921.300	-341,527.800	-2,947,678.000	-413,841.500	-464,948.000
Akaike Inf. Crit.	497,860.600	683,073.600	5,895,373.000	827,701.000	929,914.000

Note: *p<0.05;* ***p<0.0035;*** p<1e-05

Table S10. Univariate associations between rs13112358 (*NEIL3*) and cognitive outcomes after controlling for age (centered on 60 years), sex, and education. Statistics reported include unstandardized Beta estimates and p values. Asterisks indicate the significance levels at *p<0.05; **p<0.0035 (Bonferroni corrected); ***p<0.00001.

	Cognitive Measures				
SNP variants (1/2 alleles vs none)	FIQ	SDMT	MATCH	TRAIL1	TRAIL2
age_60	-0.017***	-0.271***	3.625***	0.493***	1.030***
	p = 0.000	p = 0.000	p = 0.000	p = 0.000	p = 0.000
sexMale	0.190***	0.026	-17.993***	-2.253***	-2.484***
	p = 0.000	p = 0.357	p = 0.000	p = 0.000	p = 0.000
educationSecondary	1.293***	2.073***	-25.240***	-4.524***	-13.265***
	p = 0.000	p = 0.000	p = 0.000	p = 0.000	p = 0.000
educationProf cert/dip	0.909***	1.306***	-18.430***	-3.117***	-8.693***
	p = 0.000	p = 0.000	p = 0.000	p = 0.000	p = 0.000
educationTertiary	2.241***	2.921***	-32.143***	-6.348***	-18.228***
	p = 0.000	p = 0.000	p = 0.000	p = 0.000	p = 0.000
educationUnknown	0.865***	0.814**	11.357***	-3.550***	-7.949***
	p = 0.000	p = 0.00002	p = 0.000	p = 0.00000	p = 0.000
rs131123581	0.085**	0.038	-2.767**	0.214	0.058
	p = 0.001	p = 0.537	p = 0.0002	p = 0.284	p = 0.861
rs131123582	0.098**	0.077	-4.551***	0.141	-0.235
	p = 0.00004	p = 0.192	p = 0.000	p = 0.469	p = 0.466
Constant	3.610***	16.324***	605.634***	47.018***	86.507***
	p = 0.000	p = 0.000	p = 0.000	p = 0.000	p = 0.000
Observations	120,801	115,763	481,316	101,683	101,681
Log Likelihood	-249,547.200	-342,354.700	-2,954,804.000	-414,851.900	-466,125.600
Akaike Inf. Crit.	499,112.500	684,727.400	5,909,625.000	829,721.900	932,269.200
Note:					*p<0.05;* ***p<0.0035;*** p<1e-05

Table S11. Univariate associations between rs1395479 (*NEIL3*) and cognitive outcomes after controlling for age (centered on 60 years), sex, and education. Statistics reported include unstandardized Beta estimates and p values. Asterisks indicate the significance levels at *p<0.05; **p<0.0035 (Bonferroni corrected); ***p<0.00001.

	Cognitive Measures				
SNP variants (1/2 alleles vs none)	FIQ	SDMT	MATCH	TRAIL1	TRAIL2
age_60	-0.017***	-0.271***	3.627***	0.494***	1.031***
	p = 0.000	p = 0.000	p = 0.000	p = 0.000	p = 0.000
sexMale	0.190***	0.026	-17.971***	-2.252***	-2.471***
	p = 0.000	p = 0.341	p = 0.000	p = 0.000	p = 0.000
educationSecondary	1.293***	2.066***	-25.192***	-4.479***	-13.194***
	p = 0.000	p = 0.000	p = 0.000	p = 0.000	p = 0.000
educationProf cert/dip	0.911***	1.305***	-18.369***	-3.108***	-8.638***
	p = 0.000	p = 0.000	p = 0.000	p = 0.000	p = 0.000
educationTertiary	2.242***	2.917***	-32.075***	-6.316***	-18.183***
	p = 0.000	p = 0.000	p = 0.000	p = 0.000	p = 0.000
educationUnknown	0.868***	0.807**	11.589***	-3.526***	-7.933***
	p = 0.000	p = 0.00002	p = 0.000	p = 0.00000	p = 0.000
rs13954791	0.027*	0.115**	-3.063***	-0.081	-0.323*
	p = 0.021	p = 0.0001	p = 0.000	p = 0.389	p = 0.039
rs13954792	0.011	0.077	-3.840***	-0.035	-0.258
	p = 0.622	p = 0.163	p = 0.000	p = 0.849	p = 0.388
Constant	3.686***	16.337***	603.389***	47.183***	86.487***
	p = 0.000	p = 0.000	p = 0.000	p = 0.000	p = 0.000
Observations	120,946	115,893	481,857	101,802	101,800
Log Likelihood	-249,819.500	-342,715.600	-2,957,940.000	-415,356.800	-466,678.000
Akaike Inf. Crit.	499,656.900	685,449.300	5,915,898.000	830,731.600	933,374.000

Note: *p<0.05;* ***p<0.0035;*** p<1e-05

Table S12. Univariate associations between rs34612342 (*MUTYH*) and cognitive outcomes after controlling for age (centered on 60 years), sex, and education. Statistics reported include unstandardized Beta estimates and p values. Asterisks indicate the significance levels at *p<0.05; **p<0.0035 (Bonferroni corrected); ***p<0.00001.

	Cognitive Measures				
SNP variants (1/2 alleles vs none)	FIQ	SDMT	MATCH	TRAIL1	TRAIL2
age_60	-0.017***	-0.271***	3.623***	0.494***	1.030***
	p = 0.000	p = 0.000	p = 0.000	p = 0.000	p = 0.000
sexMale	0.190***	0.025	-17.998***	-2.255***	-2.487***
	p = 0.000	p = 0.361	p = 0.000	p = 0.000	p = 0.000
educationSecondary	1.293***	2.072***	-25.220***	-4.508***	-13.230***
	p = 0.000	p = 0.000	p = 0.000	p = 0.000	p = 0.000
educationProf cert/dip	0.909***	1.308***	-18.382***	-3.117***	-8.675***
	p = 0.000	p = 0.000	p = 0.000	p = 0.000	p = 0.000
educationTertiary	2.241***	2.921***	-32.105***	-6.343***	-18.216***
	p = 0.000	p = 0.000	p = 0.000	p = 0.000	p = 0.000
educationUnknown	0.868***	0.809**	11.508***	-3.522***	-7.989***
	p = 0.000	p = 0.00002	p = 0.000	p = 0.00000	p = 0.000
rs346123421	0.101	-0.121	-3.332	1.834*	1.631
	p = 0.218	p = 0.552	p = 0.175	p = 0.007	p = 0.145
Constant	3.697***	16.384***	601.987***	47.166***	86.375***
	p = 0.000	p = 0.000	p = 0.000	p = 0.000	p = 0.000
Observations	121,050	116,001	482,379	101,897	101,895
Log Likelihood	-250,037.300	-343,059.300	-2,961,256.000	-415,733.300	-467,136.200
Akaike Inf. Crit.	500,090.700	686,134.700	5,922,528.000	831,482.500	934,288.400

Note: *p<0.05;* ***p<0.0035;*** p<1e-05

Table S13. Univariate associations between rs200165598 (*MUTYH*) and cognitive outcomes after controlling for age (centered on 60 years), sex, and education. Statistics reported include unstandardized Beta estimates and p values. Asterisks indicate the significance levels at *p<0.05; **p<0.0035 (Bonferroni corrected); ***p<0.00001.

	Cognitive Measures				
SNP variants (1/2 alleles vs none)	FIQ	SDMT	MATCH	TRAIL1	TRAIL2
age_60	-0.017***	-0.271***	3.624***	0.494***	1.030***
	p = 0.000	p = 0.000	p = 0.000	p = 0.000	p = 0.000
sexMale	0.190***	0.027	-17.997***	-2.254***	-2.487***
	p = 0.000	p = 0.335	p = 0.000	p = 0.000	p = 0.000
educationSecondary	1.292***	2.071***	-25.214***	-4.511***	-13.226***
	p = 0.000	p = 0.000	p = 0.000	p = 0.000	p = 0.000
educationProf cert/dip	0.910***	1.309***	-18.367***	-3.128***	-8.674***
	p = 0.000	p = 0.000	p = 0.000	p = 0.000	p = 0.000
educationTertiary	2.241***	2.922***	-32.095***	-6.347***	-18.212***
	p = 0.000	p = 0.000	p = 0.000	p = 0.000	p = 0.000
educationUnknown	0.867***	0.812**	11.515***	-3.551***	-7.955***
	p = 0.000	p = 0.00002	p = 0.000	p = 0.00000	p = 0.000
rs2001655981	0.143	-0.695	17.940**	1.476	4.291
	p = 0.500	p = 0.186	p = 0.004	p = 0.406	p = 0.145
Constant	3.698***	16.383***	601.955***	47.175***	86.369***
	p = 0.000	p = 0.000	p = 0.000	p = 0.000	p = 0.000
Observations	121,167	116,109	482,776	101,992	101,990
Log Likelihood	-250,294.000	-343,380.500	-2,963,676.000	-416,122.800	-467,548.200
Akaike Inf. Crit.	500,604.000	686,777.100	5,927,369.000	832,261.600	935,112.400

Note: *p<0.05;* ***p<0.0035;*** p<1e-05

Table S14. Univariate associations between rs150766139 (*NTHL1*) and cognitive outcomes after controlling for age (centered on 60 years), sex, and education. Statistics reported include unstandardized Beta estimates and p values. Asterisks indicate the significance levels at *p<0.05; **p<0.0035 (Bonferroni corrected); ***p<0.00001.

	Cognitive Measures				
SNP variants (1/2 alleles vs none)	FIQ	SDMT	MATCH	TRAIL1	TRAIL2
age_60	-0.017***	-0.271***	3.624***	0.494***	1.030***
	p = 0.000	p = 0.000	p = 0.000	p = 0.000	p = 0.000
sexMale	0.190***	0.027	-17.997***	-2.254***	-2.484***
	p = 0.000	p = 0.336	p = 0.000	p = 0.000	p = 0.000
educationSecondary	1.293***	2.070***	-25.221***	-4.512***	-13.237***
	p = 0.000	p = 0.000	p = 0.000	p = 0.000	p = 0.000
educationProf cert/dip	0.909***	1.306***	-18.379***	-3.127***	-8.679***
	p = 0.000	p = 0.000	p = 0.000	p = 0.000	p = 0.000
educationTertiary	2.241***	2.920***	-32.105***	-6.347***	-18.221***
	p = 0.000	p = 0.000	p = 0.000	p = 0.000	p = 0.000
educationUnknown	0.867***	0.810**	11.487***	-3.552***	-7.967***
	p = 0.000	p = 0.00002	p = 0.000	p = 0.00000	p = 0.000
rs1507661391	0.099	0.463	-7.418*	-0.665	-1.205
	p = 0.303	p = 0.055	p = 0.010	p = 0.410	p = 0.367
Constant	3.697***	16.382***	602.001***	47.179***	86.384***
	p = 0.000	p = 0.000	p = 0.000	p = 0.000	p = 0.000
Observations	121,151	116,093	482,736	101,976	101,974
Log Likelihood	-250,264.600	-343,326.500	-2,963,437.000	-416,065.100	-467,472.300
Akaike Inf. Crit.	500,545.100	686,669.000	5,926,890.000	832,146.100	934,960.700

Note: *p<0.05;* ***p<0.0035;*** p<1e-05

Table S15. Univariate associations between rs2516739 (*NTHL1*) and cognitive outcomes after controlling for age (centered on 60 years), sex, and education. Statistics reported include unstandardized Beta estimates and p values. Asterisks indicate the significance levels at *p<0.05; **p<0.0035 (Bonferroni corrected); ***p<0.00001.

	Cognitive Measures				
SNP variants (1/2 alleles vs none)	FIQ	SDMT	MATCH	TRAIL1	TRAIL2
age_60	-0.017***	-0.271***	3.626***	0.493***	1.030***
	p = 0.000	p = 0.000	p = 0.000	p = 0.000	p = 0.000
sexMale	0.190***	0.026	-17.995***	-2.246***	-2.476***
	p = 0.000	p = 0.342	p = 0.000	p = 0.000	p = 0.000
educationSecondary	1.294***	2.073***	-25.260***	-4.512***	-13.236***
	p = 0.000	p = 0.000	p = 0.000	p = 0.000	p = 0.000
educationProf cert/dip	0.911***	1.310***	-18.460***	-3.129***	-8.690***
	p = 0.000	p = 0.000	p = 0.000	p = 0.000	p = 0.000
educationTertiary	2.243***	2.924***	-32.170***	-6.349***	-18.221***
	p = 0.000	p = 0.000	p = 0.000	p = 0.000	p = 0.000
educationUnknown	0.868***	0.813**	11.460***	-3.549***	-7.958***
	p = 0.000	p = 0.00002	p = 0.000	p = 0.00000	p = 0.000
rs25167391	-0.003	-0.010	1.016**	-0.063	-0.051
	p = 0.790	p = 0.724	p = 0.004	p = 0.512	p = 0.747
rs25167392	-0.080**	-0.105	6.187***	0.240	0.709*
	p = 0.002	p = 0.102	p = 0.000	p = 0.254	p = 0.042
Constant	3.702***	16.391***	601.360***	47.180***	86.355***
	p = 0.000	p = 0.000	p = 0.000	p = 0.000	p = 0.000
Observations	121,041	115,984	482,186	101,878	101,876
Log Likelihood	-250,014.500	-343,000.400	-2,960,011.000	-415,627.300	-467,002.600
Akaike Inf. Crit.	500,047.100	686,018.900	5,920,041.000	831,272.600	934,023.200

Note: *p<0.05;* ***p<0.0035;*** p<1e-05

Table S16. Associations between all allele permutations of rs7402844 (protective) and rs13112358 (protective) and cognitive measures. Main associations (FIQ, MATCH) suggest an additive effect. Statistics reported include unstandardized Beta estimates and p values. Asterisks indicate the significance levels at *p<0.1; **p<0.05; ***p<0.01

	Dependent variable:				
	FIQ_0	SDMT_0	MATCH_0	TRAIL1_0	TRAIL2_0
age_60	-0.017***<br>p = 0.000	-0.271***<br>p = 0.000	3.632***<br>p = 0.000	0.493***<br>p = 0.000	1.029***<br>p = 0.000
sexMale	0.189***<br>p = 0.000	0.025<br>p = 0.372	-17.982***<br>p = 0.000	-2.256***<br>p = 0.000	-2.488***<br>p = 0.000
educationSecondary	1.293***<br>p = 0.000	2.075***<br>p = 0.000	-25.221***<br>p = 0.000	-4.533***<br>p = 0.000	-13.283***<br>p = 0.000
educationProf cert/dip	0.911***<br>p = 0.000	1.312***<br>p = 0.000	-18.477***<br>p = 0.000	-3.134***<br>p = 0.000	-8.719***<br>p = 0.000
educationTertiary	2.243***<br>p = 0.000	2.923***<br>p = 0.000	-32.210***<br>p = 0.000	-6.357***<br>p = 0.000	-18.250***<br>p = 0.000
educationUnknown	0.866***<br>p = 0.000	0.816***<br>p = 0.00002	11.055***<br>p = 0.000	-3.553***<br>p = 0.00000	-7.959***<br>p = 0.000
rs7402844_by_rs131123581x0	0.262***<br>p = 0.005	0.072<br>p = 0.752	-13.620***<br>p = 0.00000	-0.538<br>p = 0.473	-0.649<br>p = 0.601
rs7402844_by_rs131123580x1	0.265***<br>p = 0.004	0.030<br>p = 0.893	-6.853***<br>p = 0.008	-0.063<br>p = 0.933	-0.502<br>p = 0.685
rs7402844_by_rs131123581x1	0.389***<br>p = 0.00001	0.192<br>p = 0.363	-16.282***<br>p = 0.000	-0.716<br>p = 0.305	-1.803<br>p = 0.119
rs7402844_by_rs131123582x0	0.363***<br>p = 0.00005	0.249<br>p = 0.262	-16.865***<br>p = 0.000	-1.255*<br>p = 0.087	-2.790**<br>p = 0.022
rs7402844_by_rs131123580x2	0.351***<br>p = 0.0001	0.321<br>p = 0.144	-10.247***<br>p = 0.00005	-1.040<br>p = 0.152	-1.652<br>p = 0.169
rs7402844_by_rs131123582x1	0.393***<br>p = 0.00001	0.227<br>p = 0.280	-18.889***<br>p = 0.000	-0.713<br>p = 0.306	-1.787<br>p = 0.121
rs7402844_by_rs131123581x2	0.384***<br>p = 0.00001	0.214<br>p = 0.307	-18.294***<br>p = 0.000	-0.747<br>p = 0.282	-1.881<br>p = 0.102
rs7402844_by_rs131123582x2	0.410***<br>p = 0.00001	0.248<br>p = 0.235	-20.240***<br>p = 0.000	-0.711<br>p = 0.305	-2.118*<br>p = 0.065
Constant	3.311***<br>p = 0.000	16.161***<br>p = 0.000	619.969***<br>p = 0.000	47.912***<br>p = 0.000	88.289***<br>p = 0.000
Observations	120,658	115,626	480,730	101,566	101,564
Log Likelihood	-249,228.400	-341,942.400	-2,951,085.000	-414,360.400	-465,558.700
Akaike Inf. Crit.	498,486.700	683,914.700	5,902,201.000	828,750.800	931,147.400

Note: *p<0.1;* ***p<0.05;*** p<0.01

Table S17. Associations between all allele permutations of rs7402844 (protective) and rs1395479 (protective) and cognitive measures. Main associations (FIQ, MATCH) suggest a partially additive effect. Statistics reported include unstandardized Beta estimates and p values. Asterisks indicate the significance levels at *p<0.1; **p<0.05; ***p<0.01

	Dependent variable:				
	FIQ_0	SDMT_0	MATCH_0	TRAIL1_0	TRAIL2_0
age_60	-0.017***	-0.271***	3.635***	0.493***	1.031***
	p = 0.000	p = 0.000	p = 0.000	p = 0.000	p = 0.000
sexMale	0.189***	0.026	-17.970***	-2.255***	-2.475***
	p = 0.000	p = 0.355	p = 0.000	p = 0.000	p = 0.000
educationSecondary	1.293***	2.066***	-25.162***	-4.485***	-13.203***
	p = 0.000	p = 0.000	p = 0.000	p = 0.000	p = 0.000
educationProf cert/dip	0.911***	1.309***	-18.420***	-3.120***	-8.653***
	p = 0.000	p = 0.000	p = 0.000	p = 0.000	p = 0.000
educationTertiary	2.243***	2.919***	-32.132***	-6.321***	-18.198***
	p = 0.000	p = 0.000	p = 0.000	p = 0.000	p = 0.000
educationUnknown	0.869***	0.808***	11.232***	-3.531***	-7.953***
	p = 0.000	p = 0.00002	p = 0.000	p = 0.00000	p = 0.000
rs7402844_by_rs13954791x0	0.124***	0.049	-13.210***	-0.168	-1.079**
	p = 0.0001	p = 0.527	p = 0.000	p = 0.508	p = 0.011
rs7402844_by_rs13954790x1	0.129***	0.207*	-14.026***	-0.214	-1.406**
	p = 0.005	p = 0.064	p = 0.000	p = 0.559	p = 0.021
rs7402844_by_rs13954791x1	0.155***	0.146*	-16.394***	-0.278	-1.625***
	p = 0.00001	p = 0.066	p = 0.000	p = 0.288	p = 0.0002
rs7402844_by_rs13954792x0	0.159***	0.091	-16.347***	-0.224	-1.538***
	p = 0.00000	p = 0.226	p = 0.000	p = 0.367	p = 0.0002
rs7402844_by_rs13954790x2	0.117	0.084	-10.925***	-0.030	-1.489
	p = 0.178	p = 0.700	p = 0.00003	p = 0.966	p = 0.200
rs7402844_by_rs13954792x1	0.170***	0.209***	-17.828***	-0.274	-1.602***
	p = 0.00000	p = 0.007	p = 0.000	p = 0.280	p = 0.0002
rs7402844_by_rs13954791x2	0.175***	0.158	-18.322***	-0.280	-1.435**
	p = 0.0001	p = 0.153	p = 0.000	p = 0.441	p = 0.017
rs7402844_by_rs13954792x2	0.129***	0.143	-18.268***	-0.196	-1.568***
	p = 0.002	p = 0.151	p = 0.000	p = 0.549	p = 0.004
Constant	3.550***	16.267***	617.414***	47.375***	87.764***
	p = 0.000	p = 0.000	p = 0.000	p = 0.000	p = 0.000
Observations	120,802	115,755	481,271	101,685	101,683
Log Likelihood	-249,500.100	-342,303.000	-2,954,183.000	-414,871.000	-466,113.900
Akaike Inf. Crit.	499,030.100	684,636.000	5,908,396.000	829,772.000	932,257.900

Note:	*p<0.1;* ***p<0.05;*** p<0.01

Table S18. Associations between all allele permutations of rs13112358 (protective) and rs1395479 (protective) and cognitive measures. Main associations (FIQ, MATCH) suggest a partially additive effect. Statistics reported include unstandardized Beta estimates and p values. Asterisks indicate the significance levels at *p<0.1; **p<0.05; ***p<0.01

	Dependent variable:				
	FIQ_0	SDMT_0	MATCH_0	TRAIL1_0	TRAIL2_0
age_60	-0.017***	-0.271***	3.628***	0.493***	1.031***
	p = 0.000	p = 0.000	p = 0.000	p = 0.000	p = 0.000
sexMale	0.189***	0.026	-17.965***	-2.252***	-2.471***
	p = 0.000	p = 0.356	p = 0.000	p = 0.000	p = 0.000
educationSecondary	1.294***	2.067***	-25.213***	-4.492***	-13.229***
	p = 0.000	p = 0.000	p = 0.000	p = 0.000	p = 0.000
educationProf cert/dip	0.910***	1.301***	-18.431***	-3.097***	-8.653***
	p = 0.000	p = 0.000	p = 0.000	p = 0.000	p = 0.000
educationTertiary	2.243***	2.915***	-32.120***	-6.318***	-18.198***
	p = 0.000	p = 0.000	p = 0.000	p = 0.000	p = 0.000
educationUnknown	0.867***	0.809***	11.447***	-3.528***	-7.929***
	p = 0.000	p = 0.00002	p = 0.000	p = 0.00000	p = 0.000
rs1395479_by_rs131123581x0	0.049	0.116	-4.187***	0.059	-0.232
	p = 0.311	p = 0.331	p = 0.003	p = 0.880	p = 0.721
rs1395479_by_rs131123580x1	0.081**	0.044	-3.295***	0.284	0.203
	p = 0.012	p = 0.588	p = 0.0004	p = 0.282	p = 0.643
rs1395479_by_rs131123581x1	0.116***	0.140*	-6.217***	0.338	-0.088
	p = 0.001	p = 0.092	p = 0.000	p = 0.215	p = 0.846
rs1395479_by_rs131123582x0	-0.099	0.117	-5.928**	0.805	0.948
	p = 0.340	p = 0.648	p = 0.044	p = 0.338	p = 0.496
rs1395479_by_rs131123580x2	0.105***	0.074	-4.981***	0.247	-0.194
	p = 0.001	p = 0.346	p = 0.00000	p = 0.336	p = 0.649
rs1395479_by_rs131123582x1	0.148***	0.137	-6.911***	-0.233	-1.201*
	p = 0.002	p = 0.243	p = 0.00000	p = 0.544	p = 0.059
rs1395479_by_rs131123581x2	0.124***	0.196**	-7.886***	0.090	-0.504
	p = 0.0002	p = 0.014	p = 0.000	p = 0.732	p = 0.245
rs1395479_by_rs131123582x2	0.091**	0.132	-8.573***	0.430	0.145
	p = 0.023	p = 0.184	p = 0.000	p = 0.187	p = 0.788
Constant	3.595***	16.281***	607.449***	46.934***	86.533***
	p = 0.000	p = 0.000	p = 0.000	p = 0.000	p = 0.000
Observations	120,572	115,540	480,369	101,486	101,484
Log Likelihood	-249,049.500	-341,671.500	-2,948,901.000	-414,051.700	-465,226.600
Akaike Inf. Crit.	498,129.000	683,372.900	5,897,831.000	828,133.500	930,483.200

Note: *p<0.1;* ***p<0.05;*** p<0.01

Table S19. Associations between all allele permutations of rs1052133 (harmful) and rs6601606 (harmful) and cognitive measures. Main associations (FIQ, MATCH) suggest an additive effect. Statistics reported include unstandardized Beta estimates and p values. Asterisks indicate the significance levels at *p<0.1; **p<0.05; ***p<0.01

	Dependent variable:				
	FIQ_0	SDMT_0	MATCH_0	TRAIL1_0	TRAIL2_0
age_60	-0.017***	-0.271***	3.628***	0.494***	1.031***
	p = 0.000	p = 0.000	p = 0.000	p = 0.000	p = 0.000
sexMale	0.190***	0.027	-17.979***	-2.258***	-2.485***
	p = 0.000	p = 0.322	p = 0.000	p = 0.000	p = 0.000
educationSecondary	1.290***	2.073***	-25.232***	-4.515***	-13.227***
	p = 0.000	p = 0.000	p = 0.000	p = 0.000	p = 0.000
educationProf cert/dip	0.910***	1.309***	-18.421***	-3.140***	-8.697***
	p = 0.000	p = 0.000	p = 0.000	p = 0.000	p = 0.000
educationTertiary	2.241***	2.924***	-32.137***	-6.355***	-18.218***
	p = 0.000	p = 0.000	p = 0.000	p = 0.000	p = 0.000
educationUnknown	0.868***	0.813***	11.345***	-3.559***	-7.964***
	p = 0.000	p = 0.00002	p = 0.000	p = 0.00000	p = 0.000
rs6601606_by_rs10521331x0	-0.114**	-0.121	6.643***	0.217	1.297**
	p = 0.011	p = 0.277	p = 0.00000	p = 0.555	p = 0.033
rs6601606_by_rs10521330x1	-0.023*	-0.066**	0.618*	0.098	0.123
	p = 0.056	p = 0.026	p = 0.077	p = 0.313	p = 0.444
rs6601606_by_rs10521331x1	-0.214***	0.115	8.910***	-0.037	-0.360
	p = 0.0002	p = 0.403	p = 0.00000	p = 0.935	p = 0.630
rs6601606_by_rs10521332x0	-1.842***	-1.562	46.329***	3.145	-0.678
	p = 0.00002	p = 0.155	p = 0.00001	p = 0.395	p = 0.912
rs6601606_by_rs10521330x2	0.010	0.017	2.167***	0.327	0.379
	p = 0.694	p = 0.786	p = 0.004	p = 0.107	p = 0.259
rs6601606_by_rs10521332x1	-1.127***	0.015	19.484*	2.794	0.869
	p = 0.001	p = 0.987	p = 0.074	p = 0.339	p = 0.858
rs6601606_by_rs10521331x2	-0.777***	-0.203	12.191***	-1.267	-1.352
	p = 0.000	p = 0.501	p = 0.0005	p = 0.207	p = 0.417
rs6601606_by_rs10521332x2	-3.050***	-1.280	-7.555	-0.829	3.488
	p = 0.00003	p = 0.468	p = 0.656	p = 0.897	p = 0.743
Constant	3.711***	16.404***	601.425***	47.130***	86.304***
	p = 0.000	p = 0.000	p = 0.000	p = 0.000	p = 0.000
Observations	120,940	115,893	481,893	101,800	101,798
Log Likelihood	-249,776.200	-342,737.300	-2,958,201.000	-415,312.600	-466,674.000
Akaike Inf. Crit.	499,582.300	685,504.500	5,916,432.000	830,655.200	933,378.000

Note: *$p<0.1$;* ***$p<0.05$;*** $p<0.01$

Table S20. Associations between all allele permutations of rs1052133 (harmful) and rs2516739 (harmful) and cognitive measures. Statistics reported include unstandardized Beta estimates and p values. Asterisks indicate the significance levels at *p<0.1; **p<0.05; ***p<0.01

	Dependent variable:				
	FIQ_0	SDMT_0	MATCH_0	TRAIL1_0	TRAIL2_0
age_60	-0.017***	-0.271***	3.627***	0.494***	1.031***
	p = 0.000	p = 0.000	p = 0.000	p = 0.000	p = 0.000
sexMale	0.190***	0.027	-17.994***	-2.249***	-2.481***
	p = 0.000	p = 0.325	p = 0.000	p = 0.000	p = 0.000
educationSecondary	1.293***	2.074***	-25.280***	-4.515***	-13.232***
	p = 0.000	p = 0.000	p = 0.000	p = 0.000	p = 0.000
educationProf cert/dip	0.910***	1.308***	-18.489***	-3.129***	-8.695***
	p = 0.000	p = 0.000	p = 0.000	p = 0.000	p = 0.000
educationTertiary	2.243***	2.925***	-32.201***	-6.356***	-18.224***
	p = 0.000	p = 0.000	p = 0.000	p = 0.000	p = 0.000
educationUnknown	0.867***	0.812***	11.323***	-3.552***	-7.958***
	p = 0.000	p = 0.00002	p = 0.000	p = 0.00000	p = 0.000
rs2516739_by_rs10521331x0	-0.026*	-0.063	1.961***	0.029	-0.007
	p = 0.084	p = 0.101	p = 0.00002	p = 0.818	p = 0.975
rs2516739_by_rs10521330x1	-0.039***	-0.109***	1.679***	0.146	0.147
	p = 0.010	p = 0.004	p = 0.0002	p = 0.232	p = 0.469
rs2516739_by_rs10521331x1	-0.019	-0.033	1.599***	-0.004	-0.009
	p = 0.297	p = 0.464	p = 0.003	p = 0.977	p = 0.970
rs2516739_by_rs10521332x0	-0.070**	-0.160*	8.924***	0.293	0.846*
	p = 0.038	p = 0.057	p = 0.000	p = 0.287	p = 0.064
rs2516739_by_rs10521330x2	-0.068**	-0.014	4.287***	0.424*	0.133
	p = 0.030	p = 0.854	p = 0.00001	p = 0.095	p = 0.752
rs2516739_by_rs10521332x1	-0.153***	-0.147	4.503***	0.383	0.423
	p = 0.0004	p = 0.168	p = 0.0003	p = 0.271	p = 0.463
rs2516739_by_rs10521331x2	0.035	-0.019	2.193*	0.040	0.352
	p = 0.405	p = 0.851	p = 0.076	p = 0.906	p = 0.531
rs2516739_by_rs10521332x2	-0.036	-0.047	2.328	0.196	2.181
	p = 0.735	p = 0.859	p = 0.461	p = 0.819	p = 0.123
Constant	3.720***	16.429***	600.550***	47.110***	86.302***
	p = 0.000	p = 0.000	p = 0.000	p = 0.000	p = 0.000
Observations	120,867	115,819	481,511	101,733	101,731
Log Likelihood	-249,645.500	-342,517.100	-2,955,878.000	-415,028.300	-466,352.000
Akaike Inf. Crit.	499,320.900	685,064.100	5,911,786.000	830,086.600	932,734.000

Note: *p<0.1;* ***p<0.05;*** p<0.01

Table S21. Associations between all allele permutations of rs1052133 (harmful) and rs200165598 (harmful) and cognitive measures. Statistics reported include unstandardized Beta estimates and p values. Asterisks indicate the significance levels at *p<0.1; **p<0.05; ***p<0.01

	Dependent variable:				
	FIQ_0	SDMT_0	MATCH_0	TRAIL1_0	TRAIL2_0
age_60	-0.017***	-0.271***	3.625***	0.494***	1.031***
	p = 0.000	p = 0.000	p = 0.000	p = 0.000	p = 0.000
sexMale	0.190***	0.028	-18.000***	-2.256***	-2.489***
	p = 0.000	p = 0.314	p = 0.000	p = 0.000	p = 0.000
educationSecondary	1.291***	2.072***	-25.238***	-4.511***	-13.218***
	p = 0.000	p = 0.000	p = 0.000	p = 0.000	p = 0.000
educationProf cert/dip	0.909***	1.308***	-18.392***	-3.126***	-8.674***
	p = 0.000	p = 0.000	p = 0.000	p = 0.000	p = 0.000
educationTertiary	2.241***	2.924***	-32.123***	-6.351***	-18.213***
	p = 0.000	p = 0.000	p = 0.000	p = 0.000	p = 0.000
educationUnknown	0.866***	0.812***	11.432***	-3.553***	-7.955***
	p = 0.000	p = 0.00002	p = 0.000	p = 0.00000	p = 0.000
rs200165598_by_rs10521331x0	0.132	-1.066	17.229**	-0.652	0.917
	p = 0.624	p = 0.113	p = 0.030	p = 0.768	p = 0.803
rs200165598_by_rs10521330x1	-0.025**	-0.056*	0.641*	0.079	0.061
	p = 0.032	p = 0.054	p = 0.062	p = 0.409	p = 0.702
rs200165598_by_rs10521331x1	0.215	-0.075	22.268**	2.822	7.875
	p = 0.551	p = 0.933	p = 0.025	p = 0.378	p = 0.138
rs200165598_by_rs10521330x2	-0.019	0.011	2.348***	0.240	0.261
	p = 0.443	p = 0.852	p = 0.002	p = 0.228	p = 0.429
rs200165598_by_rs10521331x2	-0.620	-1.039	-2.662	23.205***	28.389**
	p = 0.574	p = 0.700	p = 0.930	p = 0.005	p = 0.039
Constant	3.708***	16.400***	601.625***	47.138***	86.337***
	p = 0.000	p = 0.000	p = 0.000	p = 0.000	p = 0.000
Observations	120,991	115,942	482,092	101,845	101,843
Log Likelihood	-249,926.500	-342,893.300	-2,959,505.000	-415,513.100	-466,888.000
Akaike Inf. Crit.	499,877.000	685,810.600	5,919,035.000	831,050.100	933,800.100
Note:					*p<0.1;* ***p<0.05;*** p<0.01

Table S22. Associations between all allele permutations of rs6601606 (harmful) and rs2516739 (harmful) and cognitive measures. Main associations (FIQ, MATCH) suggest a synergetic effect. Statistics reported include unstandardized Beta estimates and p values. Asterisks indicate the significance levels at *p<0.1; **p<0.05; ***p<0.01

	Dependent variable:				
	FIQ_0	SDMT_0	MATCH_0	TRAIL1_0	TRAIL2_0
age_60	-0.017***	-0.271***	3.630***	0.493***	1.030***
	p = 0.000	p = 0.000	p = 0.000	p = 0.000	p = 0.000
sexMale	0.190***	0.027	-17.973***	-2.245***	-2.472***
	p = 0.000	p = 0.339	p = 0.000	p = 0.000	p = 0.000
educationSecondary	1.293***	2.072***	-25.241***	-4.512***	-13.235***
	p = 0.000	p = 0.000	p = 0.000	p = 0.000	p = 0.000
educationProf cert/dip	0.913***	1.311***	-18.476***	-3.138***	-8.704***
	p = 0.000	p = 0.000	p = 0.000	p = 0.000	p = 0.000
educationTertiary	2.243***	2.923***	-32.170***	-6.352***	-18.226***
	p = 0.000	p = 0.000	p = 0.000	p = 0.000	p = 0.000
educationUnknown	0.870***	0.815***	11.322***	-3.550***	-7.961***
	p = 0.000	p = 0.00002	p = 0.000	p = 0.00000	p = 0.000
rs6601606_by_rs25167391x0	-0.241***	0.066	3.379***	-0.036	0.754
	p = 0.000	p = 0.531	p = 0.006	p = 0.919	p = 0.191
rs6601606_by_rs25167390x1	-0.009	-0.006	0.849**	-0.055	-0.023
	p = 0.442	p = 0.841	p = 0.016	p = 0.573	p = 0.885
rs6601606_by_rs25167391x1	-0.064	-0.021	9.997***	-0.541	-0.502
	p = 0.269	p = 0.885	p = 0.000	p = 0.256	p = 0.525
rs6601606_by_rs25167392x0	-1.718***	0.332	32.226***	1.910	-0.098
	p = 0.000	p = 0.673	p = 0.0002	p = 0.473	p = 0.983
rs6601606_by_rs25167390x2	-0.071***	-0.067	4.916***	0.154	0.646*
	p = 0.007	p = 0.306	p = 0.000	p = 0.470	p = 0.068
rs6601606_by_rs25167392x1	-0.300	-1.885	20.728	0.693	-3.458
	p = 0.587	p = 0.161	p = 0.131	p = 0.873	p = 0.629
rs6601606_by_rs25167391x2	-0.545***	-1.067***	42.267***	2.868**	3.095
	p = 0.0002	p = 0.004	p = 0.000	p = 0.017	p = 0.119
rs6601606_by_rs25167392x2	-3.122***	-3.536**	0.387	11.091	16.879
	p = 0.00002	p = 0.045	p = 0.988	p = 0.121	p = 0.155
Constant	3.709***	16.388***	601.242***	47.182***	86.336***
	p = 0.000	p = 0.000	p = 0.000	p = 0.000	p = 0.000
Observations	120,976	115,922	481,931	101,821	101,819
Log Likelihood	-249,839.800	-342,804.800	-2,958,334.000	-415,370.900	-466,732.600
Akaike Inf. Crit.	499,709.600	685,639.600	5,916,697.000	830,771.700	933,495.200

Note: *p<0.1;* ***p<0.05;*** p<0.01

Table S23. Associations between all allele permutations of rs6601606 (harmful) and rs200165598 (harmful) and cognitive measures. Statistics reported include unstandardized Beta estimates and p values. Asterisks indicate the significance levels at *p<0.1; **p<0.05; ***p<0.01

	Dependent variable:				
	FIQ_0	SDMT_0	MATCH_0	TRAIL1_0	TRAIL2_0
age_60	-0.017***	-0.271***	3.627***	0.493***	1.030***
	p = 0.000	p = 0.000	p = 0.000	p = 0.000	p = 0.000
sexMale	0.190***	0.027	-17.973***	-2.254***	-2.483***
	p = 0.000	p = 0.338	p = 0.000	p = 0.000	p = 0.000
educationSecondary	1.292***	2.071***	-25.207***	-4.510***	-13.224***
	p = 0.000	p = 0.000	p = 0.000	p = 0.000	p = 0.000
educationProf cert/dip	0.911***	1.310***	-18.390***	-3.138***	-8.688***
	p = 0.000	p = 0.000	p = 0.000	p = 0.000	p = 0.000
educationTertiary	2.241***	2.922***	-32.108***	-6.350***	-18.214***
	p = 0.000	p = 0.000	p = 0.000	p = 0.000	p = 0.000
educationUnknown	0.870***	0.813***	11.397***	-3.556***	-7.957***
	p = 0.000	p = 0.00002	p = 0.000	p = 0.00000	p = 0.000
rs6601606_by_rs2001655981x0	-0.192***	-0.020	7.487***	-0.047	0.449
	p = 0.000	p = 0.813	p = 0.000	p = 0.864	p = 0.323
rs6601606_by_rs2001655980x1	0.187	-0.637	15.879***	1.353	4.058
	p = 0.387	p = 0.234	p = 0.010	p = 0.457	p = 0.178
rs6601606_by_rs2001655981x1	-1.161	-2.186	103.778***	4.024	9.399
	p = 0.293	p = 0.417	p = 0.006	p = 0.627	p = 0.493
rs6601606_by_rs2001655982x0	-1.590***	-0.657	26.755***	2.449	0.579
	p = 0.000	p = 0.301	p = 0.0002	p = 0.257	p = 0.872
Constant	3.703***	16.384***	601.721***	47.175***	86.355***
	p = 0.000	p = 0.000	p = 0.000	p = 0.000	p = 0.000
Observations	121,101	116,046	482,520	101,934	101,932
Log Likelihood	-250,126.000	-343,188.300	-2,962,028.000	-415,866.400	-467,276.200
Akaike Inf. Crit.	500,274.000	686,398.700	5,924,078.000	831,754.800	934,574.500
Note:					*p<0.1; **p<0.05;** p<0.01

Table S24. Associations between all allele permutations of rs2516739 (harmful) and rs200165598 (harmful) and cognitive measures. Statistics reported include unstandardized Beta estimates and p values. Asterisks indicate the significance levels at *p<0.1; **p<0.05; ***p<0.01

	Dependent variable:				
	FIQ_0	SDMT_0	MATCH_0	TRAIL1_0	TRAIL2_0
age_60	-0.017***	-0.271***	3.626***	0.493***	1.030***
	p = 0.000	p = 0.000	p = 0.000	p = 0.000	p = 0.000
sexMale	0.190***	0.026	-17.993***	-2.246***	-2.478***
	p = 0.000	p = 0.340	p = 0.000	p = 0.000	p = 0.000
educationSecondary	1.294***	2.072***	-25.255***	-4.512***	-13.230***
	p = 0.000	p = 0.000	p = 0.000	p = 0.000	p = 0.000
educationProf cert/dip	0.911***	1.310***	-18.454***	-3.129***	-8.686***
	p = 0.000	p = 0.000	p = 0.000	p = 0.000	p = 0.000
educationTertiary	2.243***	2.923***	-32.165***	-6.350***	-18.217***
	p = 0.000	p = 0.000	p = 0.000	p = 0.000	p = 0.000
educationUnknown	0.868***	0.812***	11.468***	-3.548***	-7.951***
	p = 0.000	p = 0.00002	p = 0.000	p = 0.00000	p = 0.000
rs200165598_by_rs25167391x0	-0.038	-1.430**	19.862***	3.028	8.328**
	p = 0.892	p = 0.034	p = 0.009	p = 0.181	p = 0.027
rs200165598_by_rs25167390x1	-0.003	-0.011	1.020***	-0.061	-0.044
	p = 0.772	p = 0.702	p = 0.004	p = 0.527	p = 0.781
rs200165598_by_rs25167391x1	0.472	-0.109	16.225	-0.418	-1.086
	p = 0.184	p = 0.906	p = 0.147	p = 0.894	p = 0.834
rs200165598_by_rs25167390x2	-0.080***	-0.109*	6.199***	0.245	0.721**
	p = 0.002	p = 0.090	p = 0.000	p = 0.244	p = 0.039
rs200165598_by_rs25167391x2	-0.140	3.168	16.443	-4.211	-7.459
	p = 0.870	p = 0.129	p = 0.523	p = 0.557	p = 0.529
Constant	3.702***	16.392***	601.339***	47.178***	86.345***
	p = 0.000	p = 0.000	p = 0.000	p = 0.000	p = 0.000
Observations	121,027	115,971	482,133	101,866	101,864
Log Likelihood	-249,982.500	-342,962.700	-2,959,690.000	-415,576.400	-466,948.000
Akaike Inf. Crit.	499,988.900	685,949.300	5,919,403.000	831,176.900	933,920.000
Note:					*p<0.1;* ***p<0.05;*** p<0.01

Table S25. Associations between all allele permutations of rs7402844 (protective) and rs1052133 (harmful) and cognitive measures. Main associations (MATCH) suggest a subtractive effect. Statistics reported include unstandardized Beta estimates and p values. Asterisks indicate the significance levels at *p<0.1; **p<0.05; ***p<0.01

	Dependent variable:				
	FIQ_0	SDMT_0	MATCH_0	TRAIL1_0	TRAIL2_0
age_60	-0.017***	-0.271***	3.632***	0.493***	1.031***
	p = 0.000	p = 0.000	p = 0.000	p = 0.000	p = 0.000
sexMale	0.189***	0.027	-17.987***	-2.259***	-2.490***
	p = 0.000	p = 0.331	p = 0.000	p = 0.000	p = 0.000
educationSecondary	1.292***	2.074***	-25.230***	-4.520***	-13.233***
	p = 0.000	p = 0.000	p = 0.000	p = 0.000	p = 0.000
educationProf cert/dip	0.909***	1.312***	-18.450***	-3.141***	-8.698***
	p = 0.000	p = 0.000	p = 0.000	p = 0.000	p = 0.000
educationTertiary	2.242***	2.926***	-32.195***	-6.357***	-18.229***
	p = 0.000	p = 0.000	p = 0.000	p = 0.000	p = 0.000
educationUnknown	0.867***	0.815***	11.122***	-3.562***	-7.982***
	p = 0.000	p = 0.00002	p = 0.000	p = 0.00000	p = 0.000
rs7402844_by_rs10521331x0	0.072**	0.060	-10.421***	-0.080	-0.761*
	p = 0.018	p = 0.429	p = 0.000	p = 0.748	p = 0.064
rs7402844_by_rs10521330x1	-0.027	-0.009	-2.161*	0.074	0.311
	p = 0.546	p = 0.937	p = 0.100	p = 0.841	p = 0.611
rs7402844_by_rs10521331x1	0.063**	-0.037	-9.747***	-0.042	-0.517
	p = 0.048	p = 0.640	p = 0.000	p = 0.871	p = 0.228
rs7402844_by_rs10521332x0	0.106***	0.080	-12.868***	-0.159	-0.917**
	p = 0.0004	p = 0.277	p = 0.000	p = 0.510	p = 0.022
rs7402844_by_rs10521330x2	-0.064	0.305	-4.038	0.588	-1.789
	p = 0.483	p = 0.177	p = 0.134	p = 0.427	p = 0.145
rs7402844_by_rs10521332x1	0.070**	0.047	-11.956***	-0.054	-1.018**
	p = 0.024	p = 0.536	p = 0.000	p = 0.828	p = 0.014
rs7402844_by_rs10521331x2	0.086*	0.017	-8.179***	-0.205	-0.508
	p = 0.069	p = 0.885	p = 0.000	p = 0.596	p = 0.427
rs7402844_by_rs10521332x2	0.073*	0.084	-9.736***	0.324	-0.323
	p = 0.084	p = 0.425	p = 0.000	p = 0.345	p = 0.570
Constant	3.621***	16.332***	612.766***	47.262***	87.150***
	p = 0.000	p = 0.000	p = 0.000	p = 0.000	p = 0.000
Observations	120,860	115,816	481,560	101,739	101,737
Log Likelihood	-249,641.100	-342,512.800	-2,956,109.000	-415,077.100	-466,376.400
Akaike Inf. Crit.	499,312.100	685,055.600	5,912,248.000	830,184.300	932,782.800

Note:	*p<0.1;* ***p<0.05;*** p<0.01

Table S26. Associations between all allele permutations of rs13112358 (protective) and rs1052133 (harmful) and cognitive measures. Main associations (MATCH) suggest a subtractive effect. Statistics reported include unstandardized Beta estimates and p values. Asterisks indicate the significance levels at *p<0.1; **p<0.05; ***p<0.01

	Dependent variable:				
	FIQ_0	SDMT_0	MATCH_0	TRAIL1_0	TRAIL2_0
age_60	-0.017***	-0.271***	3.627***	0.491***	1.025***
	p = 0.000	p = 0.000	p = 0.000	p = 0.000	p = 0.000
sexMale	0.191***	0.014	-17.990***	-2.222***	-2.450***
	p = 0.000	p = 0.627	p = 0.000	p = 0.000	p = 0.000
educationSecondary	1.284***	2.049***	-25.303***	-4.589***	-13.305***
	p = 0.000	p = 0.000	p = 0.000	p = 0.000	p = 0.000
educationProf cert/dip	0.900***	1.281***	-18.651***	-3.209***	-8.604***
	p = 0.000	p = 0.000	p = 0.000	p = 0.000	p = 0.000
educationTertiary	2.239***	2.917***	-32.382***	-6.400***	-18.302***
	p = 0.000	p = 0.000	p = 0.000	p = 0.000	p = 0.000
educationUnknown	0.814***	0.690***	10.837***	-3.371***	-7.800***
	p = 0.000	p = 0.001	p = 0.000	p = 0.00000	p = 0.000
rs1052133_by_rs131123581x0	-0.111**	-0.042	1.722	0.118	-0.474
	p = 0.023	p = 0.729	p = 0.219	p = 0.766	p = 0.469
rs1052133_by_rs131123580x1	0.042	0.038	-2.266**	0.208	-0.139
	p = 0.186	p = 0.632	p = 0.016	p = 0.427	p = 0.748
rs1052133_by_rs131123582x0	-0.294***	0.159	8.083***	0.591	0.907
	p = 0.003	p = 0.508	p = 0.004	p = 0.456	p = 0.490
rs1052133_by_rs131123580x2	0.045	0.098	-3.502***	0.208	-0.354
	p = 0.148	p = 0.208	p = 0.0002	p = 0.413	p = 0.401
rs1052133_by_rs131123582x1	-0.064	0.036	1.163	0.684*	0.988
	p = 0.189	p = 0.766	p = 0.414	p = 0.083	p = 0.131
rs1052133_by_rs131123581x2	0.026	0.021	-3.360***	0.232	-0.270
	p = 0.425	p = 0.790	p = 0.0004	p = 0.377	p = 0.534
rs1052133_by_rs131123582x2	0.116***	0.108	-2.628**	0.259	-0.753
	p = 0.008	p = 0.317	p = 0.038	p = 0.463	p = 0.197
Constant	3.672***	16.351***	604.621***	46.972***	86.622***
	p = 0.000	p = 0.000	p = 0.000	p = 0.000	p = 0.000
Observations	105,127	100,728	418,004	88,457	88,455
Log Likelihood	-217,154.200	-297,883.200	-2,564,926.000	-360,884.400	-405,589.000
Akaike Inf. Crit.	434,336.400	595,794.300	5,129,881.000	721,796.800	811,206.000
Note:					*p<0.1; **p<0.05;*** p<0.01

Table S27. Associations between all allele permutations of rs1395479 (protective) and rs1052133 (harmful) and cognitive measures. Main associations (MATCH) suggest a subtractive effect. Statistics reported include unstandardized Beta estimates and p values. Asterisks indicate the significance levels at *p<0.1; **p<0.05; ***p<0.01

	Dependent variable:				
	FIQ_0	SDMT_0	MATCH_0	TRAIL1_0	TRAIL2_0
age_60	-0.017***	-0.271***	3.619***	0.493***	1.034***
	p = 0.000	p = 0.000	p = 0.000	p = 0.000	p = 0.000
sexMale	0.195***	0.031	-18.004***	-2.285***	-2.456***
	p = 0.000	p = 0.298	p = 0.000	p = 0.000	p = 0.000
educationSecondary	1.292***	2.091***	-25.705***	-4.513***	-13.222***
	p = 0.000	p = 0.000	p = 0.000	p = 0.000	p = 0.000
educationProf cert/dip	0.910***	1.322***	-18.209***	-3.159***	-8.632***
	p = 0.000	p = 0.000	p = 0.000	p = 0.000	p = 0.000
educationTertiary	2.245***	2.953***	-32.549***	-6.329***	-18.230***
	p = 0.000	p = 0.000	p = 0.000	p = 0.000	p = 0.000
educationUnknown	0.824***	0.800***	11.879***	-3.531***	-7.521***
	p = 0.000	p = 0.0001	p = 0.000	p = 0.00000	p = 0.000
rs1052133_by_rs13954791x0	-0.032**	-0.053	0.105	0.108	0.075
	p = 0.041	p = 0.179	p = 0.822	p = 0.404	p = 0.728
rs1052133_by_rs13954790x1	0.018	0.114***	-3.700***	-0.056	-0.251
	p = 0.224	p = 0.003	p = 0.000	p = 0.653	p = 0.219
rs1052133_by_rs13954792x0	-0.057*	-0.026	1.323	0.123	0.427
	p = 0.087	p = 0.750	p = 0.176	p = 0.650	p = 0.342
rs1052133_by_rs13954790x2	-0.0004	0.092	-4.076***	-0.166	-0.621
	p = 0.988	p = 0.202	p = 0.00001	p = 0.482	p = 0.113
rs1052133_by_rs13954792x1	0.038	0.195**	0.092	0.179	-0.440
	p = 0.327	p = 0.045	p = 0.937	p = 0.573	p = 0.403
rs1052133_by_rs13954791x2	-0.020	-0.00001	-3.592***	0.116	0.220
	p = 0.585	p = 1.000	p = 0.001	p = 0.696	p = 0.654
rs1052133_by_rs13954792x2	0.066	0.050	-0.880	1.217*	1.018
	p = 0.458	p = 0.819	p = 0.740	p = 0.094	p = 0.397
Constant	3.697***	16.328***	603.590***	47.173***	86.477***
	p = 0.000	p = 0.000	p = 0.000	p = 0.000	p = 0.000
Observations	104,139	99,755	415,087	87,559	87,557
Log Likelihood	-215,186.800	-295,056.600	-2,548,700.000	-357,365.400	-401,567.900
Akaike Inf. Crit.	430,401.500	590,141.200	5,097,428.000	714,758.800	803,163.700
Note:					*p<0.1;* ***p<0.05;*** p<0.01

Table S28. Associations between all allele permutations of rs6601606 (harmful) and rs7402844 (protective) and cognitive measures. Main associations (FIQ, MATCH) suggest a subtractive effect. Statistics reported include unstandardized Beta estimates and p values. Asterisks indicate the significance levels at *p<0.1; **p<0.05; ***p<0.01

	Dependent variable:				
	FIQ_0	SDMT_0	MATCH_0	TRAIL1_0	TRAIL2_0
age_60	-0.017***	-0.271***	3.643***	0.493***	1.030***
	p = 0.000	p = 0.000	p = 0.000	p = 0.000	p = 0.000
sexMale	0.190***	0.026	-17.991***	-2.270***	-2.502***
	p = 0.000	p = 0.355	p = 0.000	p = 0.000	p = 0.000
educationSecondary	1.294***	2.081***	-25.104***	-4.516***	-13.262***
	p = 0.000	p = 0.000	p = 0.000	p = 0.000	p = 0.000
educationProf cert/dip	0.916***	1.326***	-18.533***	-3.145***	-8.731***
	p = 0.000	p = 0.000	p = 0.000	p = 0.000	p = 0.000
educationTertiary	2.247***	2.934***	-32.140***	-6.363***	-18.256***
	p = 0.000	p = 0.000	p = 0.000	p = 0.000	p = 0.000
educationUnknown	0.868***	0.832***	10.942***	-3.529***	-7.916***
	p = 0.000	p = 0.00002	p = 0.000	p = 0.00000	p = 0.000
rs7402844_by_rs66016061x0	0.067***	-0.007	-8.345***	-0.071	-0.635**
	p = 0.005	p = 0.904	p = 0.000	p = 0.711	p = 0.045
rs7402844_by_rs66016060x1	-0.573***	-0.458	23.661***	1.092	0.917
	p = 0.00001	p = 0.119	p = 0.000	p = 0.264	p = 0.571
rs7402844_by_rs66016062x0	0.087***	0.037	-10.434***	-0.103	-0.904***
	p = 0.0002	p = 0.515	p = 0.000	p = 0.582	p = 0.004
rs7402844_by_rs66016060x2	-2.001***	-1.523	81.313***	9.677*	-3.887
	p = 0.001	p = 0.388	p = 0.00001	p = 0.056	p = 0.643
rs7402844_by_rs66016062x1	-0.039	0.114	-7.824***	-0.193	-0.335
	p = 0.434	p = 0.360	p = 0.00000	p = 0.637	p = 0.620
rs7402844_by_rs66016061x2	-1.176**	-0.716	16.651	1.671	-3.522
	p = 0.012	p = 0.539	p = 0.172	p = 0.687	p = 0.607
rs7402844_by_rs66016062x2	-1.546***	-0.399	-3.052	0.268	2.749
	p = 0.00001	p = 0.635	p = 0.752	p = 0.928	p = 0.571
Constant	3.625***	16.355***	610.731***	47.272***	87.144***
	p = 0.000	p = 0.000	p = 0.000	p = 0.000	p = 0.000
Observations	119,693	114,697	476,470	100,762	100,760
Log Likelihood	-247,117.700	-339,177.200	-2,924,278.000	-411,083.500	-461,812.400
Akaike Inf. Crit.	494,263.300	678,382.300	5,848,585.000	822,195.000	923,652.700
Note:					*p<0.1;* ***p<0.05;*** p<0.01

Table S29. Associations between all allele permutations of rs13112358 (protective) and rs6601606 (harmful) and cognitive measures. Main associations (FIQ, MATCH) suggest a subtractive effect. Statistics reported include unstandardized Beta estimates and p values. Asterisks indicate the significance levels at *p<0.1; **p<0.05; ***p<0.01

	Dependent variable:				
	FIQ_0	SDMT_0	MATCH_0	TRAIL1_0	TRAIL2_0
age_60	-0.017***	-0.271***	3.635***	0.494***	1.031***
	p = 0.000	p = 0.000	p = 0.000	p = 0.000	p = 0.000
sexMale	0.188***	0.024	-18.020***	-2.258***	-2.495***
	p = 0.000	p = 0.389	p = 0.000	p = 0.000	p = 0.000
educationSecondary	1.291***	2.076***	-25.134***	-4.546***	-13.305***
	p = 0.000	p = 0.000	p = 0.000	p = 0.000	p = 0.000
educationProf cert/dip	0.910***	1.312***	-18.509***	-3.133***	-8.701***
	p = 0.000	p = 0.000	p = 0.000	p = 0.000	p = 0.000
educationTertiary	2.241***	2.921***	-32.085***	-6.377***	-18.248***
	p = 0.000	p = 0.000	p = 0.000	p = 0.000	p = 0.000
educationUnknown	0.866***	0.846***	10.782***	-3.535***	-8.121***
	p = 0.000	p = 0.00001	p = 0.000	p = 0.00000	p = 0.000
rs13112358_by_rs66016061x0	0.057**	0.029	-2.654***	0.297	0.155
	p = 0.022	p = 0.643	p = 0.0003	p = 0.146	p = 0.647
rs13112358_by_rs66016060x1	-0.789***	-0.275	10.875***	1.788*	2.736*
	p = 0.000	p = 0.357	p = 0.002	p = 0.068	p = 0.091
rs13112358_by_rs66016062x0	0.067***	0.069	-4.199***	0.202	-0.155
	p = 0.006	p = 0.256	p = 0.000	p = 0.309	p = 0.639
rs13112358_by_rs66016060x2	-2.496***	0.425	35.999**	0.661	-2.895
	p = 0.00001	p = 0.734	p = 0.015	p = 0.879	p = 0.686
rs13112358_by_rs66016062x1	-0.032	0.087	0.055	0.301	0.367
	p = 0.522	p = 0.485	p = 0.970	p = 0.466	p = 0.591
rs13112358_by_rs66016061x2	-1.437***	-0.171	14.961	1.346	1.440
	p = 0.0002	p = 0.864	p = 0.168	p = 0.691	p = 0.797
rs13112358_by_rs66016062x2	-0.973**	-1.939*	24.144**	5.740	1.990
	p = 0.023	p = 0.078	p = 0.031	p = 0.122	p = 0.746
Constant	3.644***	16.331***	605.157***	46.979***	86.446***
	p = 0.000	p = 0.000	p = 0.000	p = 0.000	p = 0.000
Observations	119,477	114,497	475,657	100,574	100,572
Log Likelihood	-246,700.100	-338,615.500	-2,919,333.000	-410,384.200	-461,089.300
Akaike Inf. Crit.	493,428.100	677,259.000	5,838,695.000	820,796.400	922,206.500
Note:					*p<0.1;* ***p<0.05;*** p<0.01

Table S30. Associations between all allele permutations of rs1395479 (protective) and rs6601606 (harmful) and cognitive measures. Main associations (FIQ, MATCH) suggest a subtractive effect. Statistics reported include unstandardized Beta estimates and p values. Asterisks indicate the significance levels at *p<0.1; **p<0.05; ***p<0.01

	Dependent variable:				
	FIQ_0	SDMT_0	MATCH_0	TRAIL1_0	TRAIL2_0
age_60	-0.017***	-0.272***	3.632***	0.494***	1.031***
	p = 0.000	p = 0.000	p = 0.000	p = 0.000	p = 0.000
sexMale	0.187***	0.022	-17.994***	-2.246***	-2.448***
	p = 0.000	p = 0.423	p = 0.000	p = 0.000	p = 0.000
educationSecondary	1.291***	2.071***	-25.096***	-4.505***	-13.232***
	p = 0.000	p = 0.000	p = 0.000	p = 0.000	p = 0.000
educationProf cert/dip	0.910***	1.315***	-18.264***	-3.134***	-8.640***
	p = 0.000	p = 0.000	p = 0.000	p = 0.000	p = 0.000
educationTertiary	2.242***	2.918***	-32.039***	-6.330***	-18.225***
	p = 0.000	p = 0.000	p = 0.000	p = 0.000	p = 0.000
educationUnknown	0.872***	0.838***	11.274***	-3.545***	-8.406***
	p = 0.000	p = 0.00001	p = 0.000	p = 0.00000	p = 0.000
rs6601606_by_rs13954791 x0	-0.251***	-0.309***	12.484***	0.372	1.407**
	p = 0.00000	p = 0.006	p = 0.000	p = 0.316	p = 0.022
rs6601606_by_rs13954790 x1	0.024**	0.095***	-2.682***	-0.057	-0.280*
	p = 0.042	p = 0.002	p = 0.000	p = 0.553	p = 0.078
rs6601606_by_rs13954792 x0	-1.603***	-1.458*	30.333***	2.362	0.778
	p = 0.00000	p = 0.057	p = 0.0004	p = 0.375	p = 0.860
rs6601606_by_rs13954790 x2	0.0001	0.064	-3.413***	-0.012	-0.146
	p = 0.995	p = 0.250	p = 0.00000	p = 0.948	p = 0.629
rs6601606_by_rs13954792 x1	-1.373***	1.180	8.015	2.542	-0.120
	p = 0.004	p = 0.311	p = 0.526	p = 0.492	p = 0.985
rs6601606_by_rs13954791 x2	0.131	0.195	-5.375	-0.851	-2.871
	p = 0.317	p = 0.552	p = 0.153	p = 0.421	p = 0.101
rs6601606_by_rs13954792 x2	-4.245**	1.814	55.606*		
	p = 0.027	p = 0.697	p = 0.055		

Constant	3.695***	16.344***	602.945***	47.187***	86.476***
	p = 0.000	p = 0.000	p = 0.000	p = 0.000	p = 0.000
Observations	119,590	114,591	476,440	100,662	100,660
Log Likelihood	-246,926.600	-338,820.900	-2,924,748.000	-410,721.800	-461,409.400
Akaike Inf. Crit.	493,881.200	677,669.700	5,849,524.000	821,469.500	922,844.800
Note:					*p<0.1;* ***p<0.05;*** p<0.01

Table S31. Associations between all allele permutations of rs7402844 (protective) and rs2516739 (harmful) and cognitive measures. Main associations (FIQ, MATCH) suggest a subtractive effect. Statistics reported include unstandardized Beta estimates and p values. Asterisks indicate the significance levels at *p<0.1; **p<0.05; ***p<0.01

	Dependent variable:				
	FIQ_0	SDMT_0	MATCH_0	TRAIL1_0	TRAIL2_0
age_60	-0.017***	-0.271***	3.644***	0.493***	1.031***
	p = 0.000	p = 0.000	p = 0.000	p = 0.000	p = 0.000
sexMale	0.179***	0.020	-17.967***	-2.221***	-2.471***
	p = 0.000	p = 0.493	p = 0.000	p = 0.000	p = 0.000
educationSecondary	1.295***	2.080***	-25.063***	-4.545***	-13.428***
	p = 0.000	p = 0.000	p = 0.000	p = 0.000	p = 0.000
educationProf cert/dip	0.908***	1.334***	-18.319***	-3.164***	-8.797***
	p = 0.000	p = 0.000	p = 0.000	p = 0.000	p = 0.000
educationTertiary	2.243***	2.939***	-32.359***	-6.392***	-18.445***
	p = 0.000	p = 0.000	p = 0.000	p = 0.000	p = 0.000
educationUnknown	0.926***	0.712***	11.888***	-3.526***	-7.949***
	p = 0.000	p = 0.0005	p = 0.000	p = 0.00000	p = 0.000
rs7402844_by_rs25167391x0	0.068**	-0.077	-5.470***	0.099	-0.236
	p = 0.024	p = 0.304	p = 0.000	p = 0.686	p = 0.557
rs7402844_by_rs25167390x1	-0.024	-0.129	6.377***	0.589	0.570
	p = 0.593	p = 0.254	p = 0.00001	p = 0.111	p = 0.348
rs7402844_by_rs25167392x0	0.092***	-0.010	-7.270***	0.167	-0.534
	p = 0.002	p = 0.892	p = 0.000	p = 0.482	p = 0.173
rs7402844_by_rs25167390x2	-0.191**	-0.619***	30.685***	1.075	3.975***
	p = 0.048	p = 0.010	p = 0.000	p = 0.176	p = 0.003
rs7402844_by_rs25167392x1	0.081***	-0.051	-6.851***	0.027	-0.490
	p = 0.008	p = 0.499	p = 0.000	p = 0.914	p = 0.230
rs7402844_by_rs25167391x2	-0.071	-0.291**	2.568*	1.043***	1.084*
	p = 0.144	p = 0.017	p = 0.068	p = 0.009	p = 0.097
rs7402844_by_rs25167392x2	0.072*	0.033	-6.677***	-0.189	-0.684
	p = 0.100	p = 0.763	p = 0.00000	p = 0.596	p = 0.243
Constant	3.630***	16.419***	607.491***	47.068***	86.926***
	p = 0.000	p = 0.000	p = 0.000	p = 0.000	p = 0.000
Observations	105,327	100,877	419,553	88,622	88,620
Log Likelihood	-217,449.700	-298,417.300	-2,574,697.000	-361,587.000	-405,720.100
Akaike Inf. Crit.	434,927.500	596,862.500	5,149,421.000	723,202.000	811,468.100
Note:					*p<0.1;* ***p<0.05;*** p<0.01

Table S32. Associations between all allele permutations of rs13112358 (protective) and rs2516739 (harmful) and cognitive measures. Main associations (FIQ, MATCH) suggest a subtractive effect. Statistics reported include unstandardized Beta estimates and p values. Asterisks indicate the significance levels at *p<0.1; **p<0.05; ***p<0.01

	Dependent variable:				
	FIQ_0	SDMT_0	MATCH_0	TRAIL1_0	TRAIL2_0
age_60	-0.017***	-0.273***	3.628***	0.496***	1.032***
	p = 0.000	p = 0.000	p = 0.000	p = 0.000	p = 0.000
sexMale	0.187***	0.033	-18.015***	-2.234***	-2.538***
	p = 0.000	p = 0.270	p = 0.000	p = 0.000	p = 0.000
educationSecondary	1.302***	2.057***	-24.975***	-4.503***	-13.076***
	p = 0.000	p = 0.000	p = 0.000	p = 0.000	p = 0.000
educationProf cert/dip	0.921***	1.295***	-18.363***	-3.125***	-8.513***
	p = 0.000	p = 0.000	p = 0.000	p = 0.000	p = 0.000
educationTertiary	2.248***	2.921***	-31.857***	-6.383***	-18.153***
	p = 0.000	p = 0.000	p = 0.000	p = 0.000	p = 0.000
educationUnknown	0.889***	0.795***	12.304***	-3.741***	-8.041***
	p = 0.000	p = 0.0001	p = 0.000	p = 0.00000	p = 0.000
rs2516739_by_rs131123581 x0	0.031	-0.130	-0.582	-0.080	-0.230
	p = 0.531	p = 0.286	p = 0.683	p = 0.842	p = 0.729
rs2516739_by_rs131123580 x1	0.101***	0.024	-3.150***	0.157	-0.035
	p = 0.002	p = 0.761	p = 0.001	p = 0.537	p = 0.934
rs2516739_by_rs131123582 x0	0.014	0.659**	8.754***	-1.322	-0.894
	p = 0.894	p = 0.014	p = 0.004	p = 0.129	p = 0.536
rs2516739_by_rs131123580 x2	0.115***	0.078	-5.043***	0.041	-0.402
	p = 0.0002	p = 0.304	p = 0.000	p = 0.870	p = 0.327
rs2516739_by_rs131123582 x1	-0.004	-0.061	4.224***	0.398	0.485
	p = 0.939	p = 0.625	p = 0.004	p = 0.328	p = 0.472
rs2516739_by_rs131123581 x2	0.108***	0.065	-3.676***	0.028	-0.381
	p = 0.001	p = 0.405	p = 0.0001	p = 0.915	p = 0.370
rs2516739_by_rs131123582 x2	0.040	-0.120	0.034	0.446	0.595
	p = 0.366	p = 0.270	p = 0.979	p = 0.213	p = 0.316
Constant	3.594***	16.338***	605.202***	47.113***	86.524***

	p = 0.000	p = 0.000	p = 0.000	p = 0.000	p = 0.000
Observations	105,791	101,379	420,811	89,058	89,056
Log Likelihood	-218,453.400	-299,874.000	-2,582,947.000	-363,330.400	-408,339.800
Akaike Inf. Crit.	436,934.900	599,775.900	5,165,922.000	726,688.800	816,707.600
Note:					*p<0.1;* ***p<0.05;*** p<0.01

Table S33. Associations between all allele permutations of rs1395479 (protective) and rs2516739 (harmful) and cognitive measures. Statistics reported include unstandardized Beta estimates and p values. Asterisks indicate the significance levels at *p<0.1; **p<0.05; ***p<0.01

	Dependent variable:				
	FIQ_0	SDMT_0	MATCH_0	TRAIL1_0	TRAIL2_0
age_60	-0.017***	-0.271***	3.606***	0.493***	1.029***
	p = 0.000	p = 0.000	p = 0.000	p = 0.000	p = 0.000
sexMale	0.190***	0.035	-17.891***	-2.271***	-2.487***
	p = 0.000	p = 0.239	p = 0.000	p = 0.000	p = 0.000
educationSecondary	1.285***	2.078***	-25.636***	-4.471***	-13.134***
	p = 0.000	p = 0.000	p = 0.000	p = 0.000	p = 0.000
educationProf cert/dip	0.894***	1.283***	-18.215***	-3.003***	-8.369***
	p = 0.000	p = 0.000	p = 0.000	p = 0.000	p = 0.000
educationTertiary	2.232***	2.937***	-32.422***	-6.272***	-18.149***
	p = 0.000	p = 0.000	p = 0.000	p = 0.000	p = 0.000
educationUnknown	0.846***	0.673***	12.813***	-3.080***	-7.336***
	p = 0.000	p = 0.001	p = 0.000	p = 0.00001	p = 0.000
rs1395479_by_rs25167391x0	0.010	0.132***	-0.855*	-0.083	-0.245
	p = 0.482	p = 0.0004	p = 0.052	p = 0.493	p = 0.221
rs1395479_by_rs25167390x1	-0.016	0.023	2.770***	-0.108	-0.066
	p = 0.324	p = 0.562	p = 0.000	p = 0.408	p = 0.760
rs1395479_by_rs25167392x0	-0.011	0.081	-1.628*	-0.086	-0.240
	p = 0.697	p = 0.254	p = 0.054	p = 0.711	p = 0.533
rs1395479_by_rs25167390x2	-0.143***	-0.193**	12.571***	0.386	1.281***
	p = 0.00004	p = 0.026	p = 0.000	p = 0.174	p = 0.007
rs1395479_by_rs25167392x1	0.043	0.099	-2.815**	-0.024	-0.395
	p = 0.238	p = 0.281	p = 0.011	p = 0.937	p = 0.426
rs1395479_by_rs25167391x2	0.045	0.189*	-4.006***	-0.078	-0.582
	p = 0.280	p = 0.068	p = 0.002	p = 0.818	p = 0.300
rs1395479_by_rs25167392x2	-0.198**	-0.173	-4.979*	0.294	1.518
	p = 0.040	p = 0.474	p = 0.083	p = 0.706	p = 0.238
Constant	3.708***	16.328***	601.885***	47.165***	86.372***
	p = 0.000	p = 0.000	p = 0.000	p = 0.000	p = 0.000
Observations	104,882	100,532	418,514	88,204	88,202
Log Likelihood	-216,634.300	-297,348.900	-2,571,076.000	-359,820.400	-404,301.100
Akaike Inf. Crit.	433,296.700	594,725.800	5,142,179.000	719,668.700	808,630.300
Note:					*p<0.1;* ***p<0.05;*** p<0.01

Table S34. Associations between all allele permutations of rs7402844 (protective) and rs200165598 (harmful) and cognitive measures. Statistics reported include unstandardized Beta estimates and p values. Asterisks indicate the significance levels at *p<0.1; **p<0.05; ***p<0.01

	Dependent variable:				
	FIQ_0	SDMT_0	MATCH_0	TRAIL1_0	TRAIL2_0
age_60	-0.017***	-0.271***	3.631***	0.493***	1.030***
	p = 0.000	p = 0.000	p = 0.000	p = 0.000	p = 0.000
sexMale	0.189***	0.026	-17.973***	-2.257***	-2.491***
	p = 0.000	p = 0.346	p = 0.000	p = 0.000	p = 0.000
educationSecondary	1.292***	2.073***	-25.196***	-4.521***	-13.240***
	p = 0.000	p = 0.000	p = 0.000	p = 0.000	p = 0.000
educationProf cert/dip	0.910***	1.315***	-18.427***	-3.145***	-8.706***
	p = 0.000	p = 0.000	p = 0.000	p = 0.000	p = 0.000
educationTertiary	2.242***	2.925***	-32.168***	-6.359***	-18.231***
	p = 0.000	p = 0.000	p = 0.000	p = 0.000	p = 0.000
educationUnknown	0.868***	0.815***	11.206***	-3.561***	-7.978***
	p = 0.000	p = 0.00002	p = 0.000	p = 0.00000	p = 0.000
rs7402844_by_rs2001655981x0	0.085***	0.010	-9.009***	-0.140	-0.679**
	p = 0.0003	p = 0.859	p = 0.000	p = 0.459	p = 0.030
rs7402844_by_rs2001655980x1	1.507***	-0.079	43.057**	-3.785	-4.263
	p = 0.007	p = 0.956	p = 0.030	p = 0.404	p = 0.570
rs7402844_by_rs2001655982x0	0.107***	0.056	-11.327***	-0.165	-0.940***
	p = 0.00001	p = 0.317	p = 0.000	p = 0.371	p = 0.003
rs7402844_by_rs2001655982x1	-0.030	-0.487	4.907	5.818**	8.696*
	p = 0.930	p = 0.544	p = 0.548	p = 0.043	p = 0.067
Constant	3.606***	16.346***	611.672***	47.327***	87.165***
	p = 0.000	p = 0.000	p = 0.000	p = 0.000	p = 0.000
Observations	120,985	115,935	482,065	101,843	101,841
Log Likelihood	-249,902.600	-342,863.500	-2,959,195.000	-415,512.200	-466,841.500
Akaike Inf. Crit.	499,827.200	685,748.900	5,918,411.000	831,046.400	933,705.000
Note:					*p<0.1; **p<0.05;*** p<0.01

Table S35. Associations between all allele permutations of rs13112358 (protective) and rs200165598 (harmful) and cognitive measures. Statistics reported include unstandardized Beta estimates and p values. Asterisks indicate the significance levels at *p<0.1; **p<0.05; ***p<0.01

	Dependent variable:				
	FIQ_0	SDMT_0	MATCH_0	TRAIL1_0	TRAIL2_0
age_60	-0.017***	-0.271***	3.625***	0.493***	1.030***
	p = 0.000	p = 0.000	p = 0.000	p = 0.000	p = 0.000
sexMale	0.189***	0.026	-17.991***	-2.252***	-2.485***
	p = 0.000	p = 0.353	p = 0.000	p = 0.000	p = 0.000
educationSecondary	1.291***	2.073***	-25.214***	-4.518***	-13.242***
	p = 0.000	p = 0.000	p = 0.000	p = 0.000	p = 0.000
educationProf cert/dip	0.908***	1.306***	-18.412***	-3.112***	-8.672***
	p = 0.000	p = 0.000	p = 0.000	p = 0.000	p = 0.000
educationTertiary	2.240***	2.920***	-32.121***	-6.343***	-18.206***
	p = 0.000	p = 0.000	p = 0.000	p = 0.000	p = 0.000
educationUnknown	0.864***	0.814***	11.377***	-3.544***	-7.926***
	p = 0.000	p = 0.00002	p = 0.000	p = 0.00000	p = 0.000
rs200165598_by_rs131123581 x0	0.591	0.052	21.473	-3.860	-3.077
	p = 0.414	p = 0.979	p = 0.459	p = 0.547	p = 0.772
rs200165598_by_rs131123580 x1	0.085***	0.038	-2.772***	0.210	0.056
	p = 0.0005	p = 0.532	p = 0.0001	p = 0.295	p = 0.867
rs200165598_by_rs131123580 x2	0.099***	0.079	-4.561***	0.135	-0.243
	p = 0.00004	p = 0.182	p = 0.000	p = 0.488	p = 0.452
rs200165598_by_rs131123581 x2	0.357	-1.572**	16.426**	4.530*	9.452**
	p = 0.228	p = 0.032	p = 0.038	p = 0.070	p = 0.023
Constant	3.610***	16.324***	605.608***	47.017***	86.489***
	p = 0.000	p = 0.000	p = 0.000	p = 0.000	p = 0.000
Observations	120,756	115,718	481,139	101,644	101,642
Log Likelihood	-249,460.400	-342,227.500	-2,953,692.000	-414,690.000	-465,943.300
Akaike Inf. Crit.	498,942.900	684,477.000	5,907,405.000	829,401.900	931,908.600
Note:					*p<0.1;* ***p<0.05;*** p<0.01

Table S36. Associations between all allele permutations of rs1395479 (protective) and rs200165598 (harmful) and cognitive measures. Statistics reported include unstandardized Beta estimates and p values. Asterisks indicate the significance levels at *p<0.1; **p<0.05; ***p<0.01

	Dependent variable:				
	FIQ_0	SDMT_0	MATCH_0	TRAIL1_0	TRAIL2_0
age_60	-0.017***	-0.271***	3.627***	0.494***	1.031***
	p = 0.000	p = 0.000	p = 0.000	p = 0.000	p = 0.000
sexMale	0.189***	0.027	-17.965***	-2.251***	-2.470***
	p = 0.000	p = 0.337	p = 0.000	p = 0.000	p = 0.000
educationSecondary	1.293***	2.065***	-25.178***	-4.480***	-13.180***
	p = 0.000	p = 0.000	p = 0.000	p = 0.000	p = 0.000
educationProf cert/dip	0.910***	1.305***	-18.352***	-3.106***	-8.622***
	p = 0.000	p = 0.000	p = 0.000	p = 0.000	p = 0.000
educationTertiary	2.242***	2.917***	-32.068***	-6.317***	-18.172***
	p = 0.000	p = 0.000	p = 0.000	p = 0.000	p = 0.000
educationUnknown	0.868***	0.806***	11.609***	-3.525***	-7.919***
	p = 0.000	p = 0.00002	p = 0.000	p = 0.00000	p = 0.000
rs1395479_by_rs2001655981x0	0.027**	0.116***	-3.071***	-0.083	-0.326**
	p = 0.020	p = 0.0001	p = 0.000	p = 0.382	p = 0.037
rs1395479_by_rs2001655980x1	0.358	-0.225	13.107	0.140	2.370
	p = 0.177	p = 0.734	p = 0.101	p = 0.951	p = 0.523
rs1395479_by_rs2001655982x0	0.012	0.076	-3.852***	-0.035	-0.252
	p = 0.596	p = 0.169	p = 0.000	p = 0.846	p = 0.399
rs1395479_by_rs2001655982x1	-1.138	0.597	12.262	5.848	-1.331
	p = 0.234	p = 0.825	p = 0.609	p = 0.480	p = 0.923
Constant	3.686***	16.338***	603.366***	47.182***	86.473***
	p = 0.000	p = 0.000	p = 0.000	p = 0.000	p = 0.000
Observations	120,907	115,855	481,682	101,770	101,768
Log Likelihood	-249,734.000	-342,612.000	-2,956,831.000	-415,228.700	-466,532.600
Akaike Inf. Crit.	499,490.000	685,246.000	5,913,684.000	830,479.400	933,087.200
Note:					*p<0.1; **p<0.05;** p<0.01

Table S37. Characteristics of the Protect (sum of all significant protective SNPs), Harm (sum of all significant harmful SNPs), and Risk (Harm – Protect + 6) indexes.

Indexes	N	Mean	St. Dev.	Min	Max
Protect	481,340	3.682	0.884	0	6
Harm	485,468	0.839	0.717	0	4
Risk	478,875	3.157	1.146	0	10

Table S38. Associations between the Risk index, including its two- and three-way interactions with age category (middle-age > 60 years vs older-age >= 60 years) and sex, and brain volume as outcome. Statistics reported include unstandardized Beta estimates and p values. Asterisks indicate the significance levels at *p<0.1; **p<0.05; ***p<0.01

	Brain Volumes				
	LHC	RHC	GM	WM	WMH
age_catolder-age	-168.927***	-178.551***	-8,800.158***	-3,125.275***	1,780.570***
	p = 0.000	p = 0.000	p = 0.000	p = 0.00001	p = 0.000
sexMale	-2.721	-29.966***	2,803.444***	-4,776.379***	-87.385
	p = 0.790	p = 0.006	p = 0.00002	p = 0.000	p = 0.308
educationSecondary	28.002***	24.852***	2,221.272***	-883.023*	-220.326***
	p = 0.0001	p = 0.001	p = 0.00000	p = 0.052	p = 0.0002
educationProf cert/dip	15.305*	13.815	971.352*	-540.795	-81.374
	p = 0.058	p = 0.106	p = 0.059	p = 0.303	p = 0.231
educationTertiary	28.897***	22.132***	2,555.103***	-2,297.303***	-260.747***
	p = 0.00003	p = 0.003	p = 0.000	p = 0.00000	p = 0.00001
educationUnknown	9.540	-0.248	1,093.552	-2,346.023*	-102.172
	p = 0.630	p = 0.991	p = 0.386	p = 0.069	p = 0.540
risk	-1.156	-2.328	14.233	-155.024	11.234
	p = 0.583	p = 0.296	p = 0.916	p = 0.258	p = 0.527
ICV_2	2.055***	2.181***	464.469***	466.549***	3.698***
	p = 0.000	p = 0.000	p = 0.000	p = 0.000	p = 0.000
age_catolder-age:risk	5.469*	8.315**	284.274	188.111	-12.034
	p = 0.088	p = 0.014	p = 0.164	p = 0.366	p = 0.655
sexMale:risk	1.450	6.099**	166.249	72.153	-25.334
	p = 0.616	p = 0.046	p = 0.366	p = 0.701	p = 0.297
age_catolder-age:sexMale:risk	-7.938***	-13.799***	-690.819***	-216.888*	94.752***
	p = 0.0001	p = 0.000	p = 0.00000	p = 0.090	p = 0.000
Constant	1,501.005***	1,540.791***	103,913.100***	-79,827.610***	-2,579.071***
	p = 0.000	p = 0.000	p = 0.000	p = 0.000	p = 0.000
Observations	38,376	38,376	38,376	38,376	38,376
Log Likelihood	-274,714.100	-276,832.500	-434,135.300	-434,922.200	-356,459.800
Akaike Inf. Crit.	549,452.200	553,689.000	868,294.500	869,868.400	712,943.600
Note:					*p<0.1;* ***p<0.05;*** p<0.01

Table S39. Associations between latent classes of individuals who cluster together based on the latent class analysis (2-class model), including two- and three-way interactions with age category (middle-age > 60 years vs older-age >= 60 years) and sex, and cognitive measures. Statistics reported include unstandardized Beta estimates and p values. Asterisks indicate the significance levels at *p<0.1; **p<0.05; ***p<0.01

	Dependent variable:				
	FIQ_0	SDMT_0	MATCH_0	TRAIL1_0	TRAIL2_0
age_catolder-age	-0.221***	-3.660***	50.892***	6.554***	14.188***
	p = 0.000	p = 0.000	p = 0.000	p = 0.000	p = 0.000
sexMale	0.238***	-0.155***	-15.631***	-2.083***	-2.067***
	p = 0.000	p = 0.00005	p = 0.000	p = 0.000	p = 0.000
educationSecondary	1.300***	2.449***	-31.031***	-5.150***	-14.463***
	p = 0.000	p = 0.000	p = 0.000	p = 0.000	p = 0.000
educationProf cert/dip	0.913***	1.462***	-20.301***	-3.374***	-9.127***
	p = 0.000	p = 0.000	p = 0.000	p = 0.000	p = 0.000
educationTertiary	2.248***	3.300***	-37.459***	-6.976***	-19.411***
	p = 0.000	p = 0.000	p = 0.000	p = 0.000	p = 0.000
educationUnknown	0.873***	1.038***	8.107***	-3.920***	-8.648***
	p = 0.000	p = 0.00000	p = 0.00000	p = 0.000	p = 0.000
c2bin_r_model2	-0.156***	-0.075	5.774***	0.069	0.558
	p = 0.0004	p = 0.506	p = 0.00003	p = 0.848	p = 0.352
age_catolder-age:c2bin_r_model2	0.139*	0.277	-4.694**	-0.778	-2.024*
	p = 0.064	p = 0.151	p = 0.030	p = 0.217	p = 0.053
sexMale:c2bin_r_model2	0.075	-0.049	-2.045	-0.212	0.266
	p = 0.280	p = 0.784	p = 0.324	p = 0.707	p = 0.777
age_catolder-age:sexMale:c2bin_r_model1	-0.119***	0.341***	-5.746***	-0.227	-0.736**
	p = 0.00000	p = 0.000	p = 0.000	p = 0.236	p = 0.021
age_catolder-age:sexMale:c2bin_r_model2	-0.248**	0.098	-1.015	1.224	1.164
	p = 0.022	p = 0.724	p = 0.744	p = 0.170	p = 0.433
Constant	3.852***	18.581***	571.482***	43.141***	77.668***
	p = 0.000	p = 0.000	p = 0.000	p = 0.000	p = 0.000
Observations	121,181	116,122	482,829	102,004	102,002
Log Likelihood	-250,291.200	-346,937.900	-2,968,877.000	-417,233.600	-469,085.500
Akaike Inf. Crit.	500,606.500	693,899.900	5,937,778.000	834,491.200	938,194.900
Note:					*p<0.1;* **p<0.05;** p<0.01

Table S40. Associations between latent classes of individuals who cluster together based on the latent class analysis (2-class model), including two- and three-way interactions with age category (middle-age > 60 years vs older-age >= 60 years) and sex, and brain outcomes. Statistics reported include unstandardized Beta estimates and p values. Asterisks indicate the significance levels at *p<0.1; **p<0.05; ***p<0.01

	Dependent variable:				
	LHC_2	RHC_2	GM_2	WM_2	WMH_2
age_catolder-age	-150.932***	-150.369***	-7,772.356***	-2,494.793***	1,739.109***
	p = 0.000	p = 0.000	p = 0.000	p = 0.000	p = 0.000
sexMale	2.810	-10.476**	3,368.015***	-4,449.808***	-157.022***
	p = 0.540	p = 0.031	p = 0.000	p = 0.000	p = 0.00005
educationSecondary	27.841***	25.391***	2,273.857***	-936.933**	-221.227***
	p = 0.0001	p = 0.001	p = 0.00000	p = 0.037	p = 0.0002
educationProf cert/dip	15.102*	14.842*	947.255*	-523.906	-88.766
	p = 0.060	p = 0.080	p = 0.064	p = 0.314	p = 0.188
educationTertiary	28.637***	22.690***	2,577.088***	-2,323.836***	-257.703***
	p = 0.00003	p = 0.002	p = 0.000	p = 0.00000	p = 0.00001
educationUnknown	6.213	0.172	946.825	-2,300.492*	-53.824
	p = 0.751	p = 0.994	p = 0.448	p = 0.071	p = 0.744
c2bin_r_model2	1.650	-4.723	653.709	-215.053	102.670
	p = 0.892	p = 0.712	p = 0.396	p = 0.784	p = 0.313
ICV_2	2.054***	2.181***	464.563***	466.311***	3.699***
	p = 0.000	p = 0.000	p = 0.000	p = 0.000	p = 0.000
age_catolder-age:c2bin_r_model2	4.246	14.239	-382.252	1,068.916	-48.055
	p = 0.859	p = 0.571	p = 0.801	p = 0.489	p = 0.810
sexMale:c2bin_r_model2	0.211	20.184	734.100	-1,337.033	-229.513
	p = 0.991	p = 0.300	p = 0.532	p = 0.264	p = 0.139
age_catolder-age:sexMale:c2bin_r_model1	-25.138***	-45.197***	-2,331.395***	-845.555*	316.701***
	p = 0.0004	p = 0.000	p = 0.00000	p = 0.062	p = 0.00000
age_catolder-age:sexMale:c2bin_r_model2	-40.601	-82.005**	-3,504.845*	-805.560	309.256
	p = 0.220	p = 0.020	p = 0.097	p = 0.708	p = 0.267
Constant	1,497.863***	1,531.517***	103,762.900***	-80,014.410***	-2,550.957***
	p = 0.000	p = 0.000	p = 0.000	p = 0.000	p = 0.000
Observations	39,061	39,061	39,061	39,061	39,061
Log Likelihood	-279,639.800	-281,822.300	-441,884.300	-442,680.900	-362,832.800
Akaike Inf. Crit.	559,305.600	563,670.600	883,794.500	885,387.900	725,691.600
Note:					*p<0.1;* **p<0.05;** p<0.01

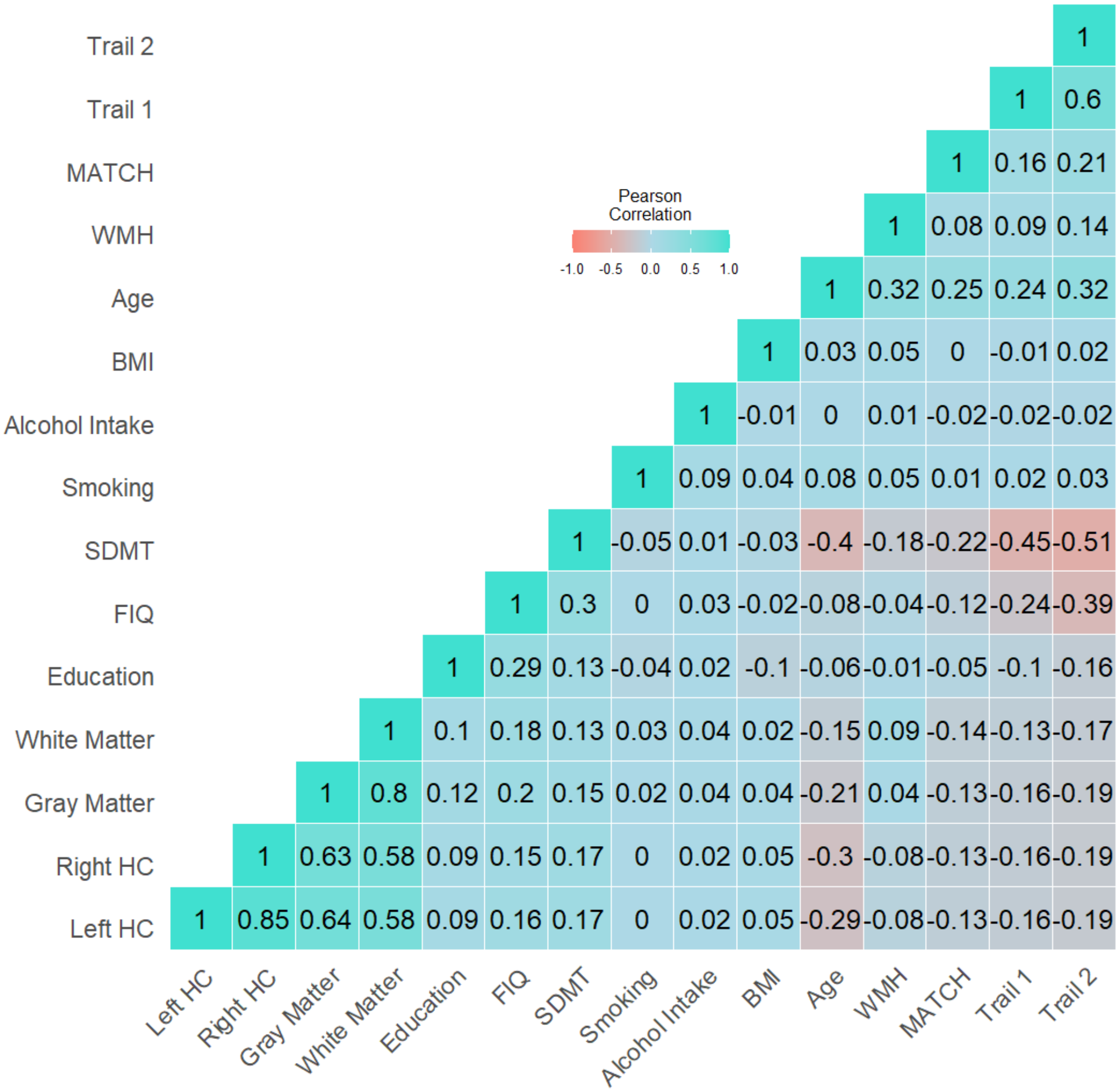

Figure S1. Bivariate Pearson correlations between outcome variables and covariates.

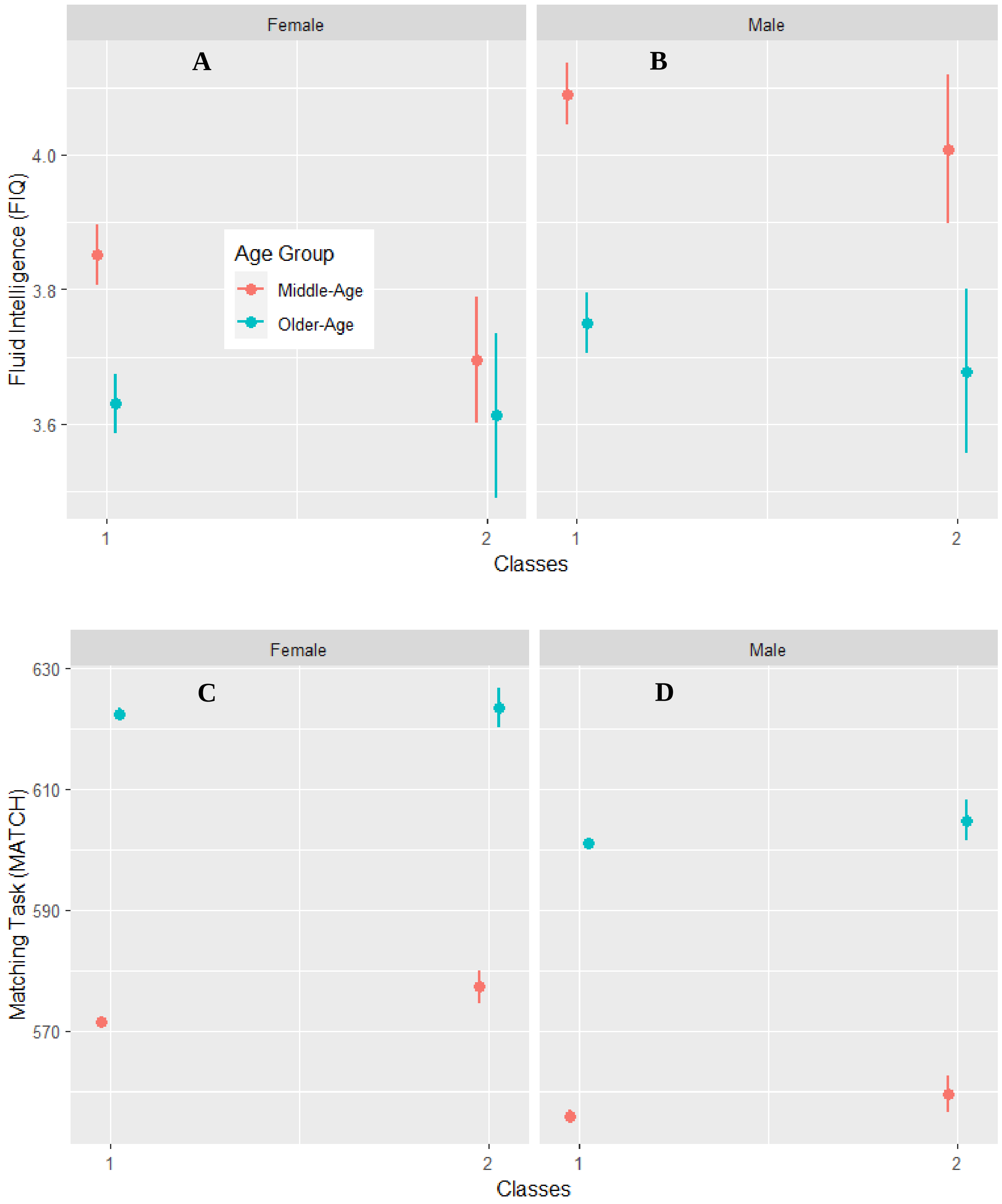

Figure S2. Depiction of the significant three-way interaction between latent classes, age and sex predicting cognitive measures including FIQ (panels A & B), and MATCH (panels C & D). Class membership was primarily based on three SNPs (*NEIL3*: rs13112358, rs13112390, rs10013040) which were present in the class 2 but either not or to a much lesser extent in class 1. For FIQ the significant interaction indicates that performance is substantially lower in middle-age women belonging to class 1 than for older-age women belonging to class 2 or men. For MATCH the significant interaction indicates that response time is substantially higher for older-age women belonging to class 1 than for older-age women belonging to class 2. Error bars indicate 95% confidence interval.

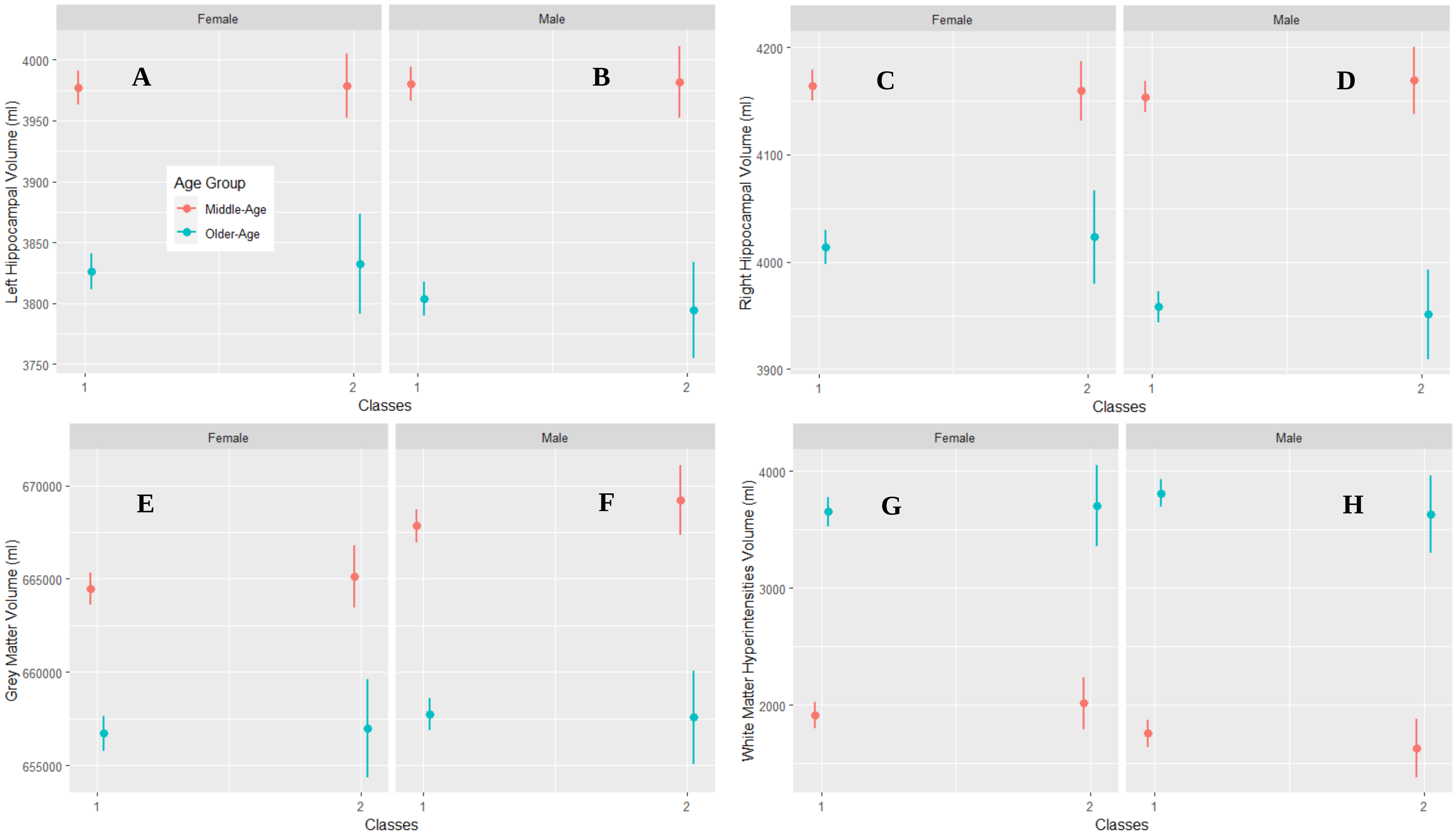

Figure S3. Depiction of the significant three-way interaction between latent classes, age and sex predicting brain measures including the left hippocampus (LHC; panels A & B), the right hippocampus (RHC; panels C & D), total grey matter (GM; panels E & F), and white matter hyperintensities (WMH; panels G & H). For LHC the significant interaction indicates that volumes were substantially larger in older-age women belonging to class 1 than for older-age men belonging to class 1, whereas they did not significantly differ between older-age women of class 2

and older-age men of class 1 & 2, or between middle-age women and men of any class. For RHC the significant interaction indicates that volumes were substantially larger in older-age women belonging to class 1 & 2 than in middle-age men belonging to class 1 & 2, whereas they did not significantly differ between middle-age women and middle-age men of any class. For GM the significant interaction indicates that volumes were substantially smaller in middle-age women belonging to class 1 & 2 than in middle-age men belonging to class 1 & 2, whereas they did not significantly differ between older-age women and older-age men of any class. For WMH the significant interaction indicates that volumes tended to be larger in class 2 than class 1 for middle-age and older-age women, whereas the opposite trend was seen in men. Error bars indicate 95% confidence interval.

Supplementary Methods

Latent class analysis

Two-class model

Conditional item response (column) probabilities,
 by outcome variable, for each class (row)

```
$rs1052133bin
            0      1
class 1:  0.5774 0.4226
class 2:  0.5901 0.4099

$rs104893751bin
            0      1
class 1:  0.9919 0.0081
class 2:  0.9918 0.0082

$rs7402844bin
            0      1
class 1:  0.0790 0.9210
class 2:  0.0682 0.9318

$rs6601606bin
            0      1
class 1:  0.9597 0.0403
class 2:  0.9701 0.0299

$rs10013040bin
            0      1
class 1:  0.6866 0.3134
class 2:  0.5959 0.4041

$rs13112390bin
            0      1
class 1:  0.7549 0.2451
class 2:  0.0000 1.0000

$rs13112358bin
            0      1
class 1:  0.9945 0.0055
class 2:  0.0000 1.0000

$rs1395479bin
            0      1
class 1:  0.5932 0.4068
class 2:  0.5471 0.4529

$rs34612342bin
            0      1
class 1:  0.9960 0.0040
class 2:  0.9956 0.0044

$rs200165598bin
            0     1
class 1:  0.9995 5e-04
class 2:  0.9993 7e-04

$rs200495564bin
        0 1
class 1:  1 0
class 2:  1 0

$rs2516739bin
            0      1
class 1:  0.6198 0.3802
class 2:  0.6069 0.3931

Estimated class population shares
 0.061 0.939

Predicted class memberships (by modal posterior prob.)
 0.0608 0.9392
```

==
Fit for 2 latent classes:
==
number of observations: 488013
number of fully observed cases: 478875
number of estimated parameters: 25
residual degrees of freedom: 4070
maximum log-likelihood: -1676240

AIC(2): 3352530
BIC(2): 3352807
G^2(2): 8351.124 (Likelihood ratio/deviance statistic)
X^2(2): 9070.049 (Chi-square goodness of fit)

Model 1: llik = -1676240 ... best llik = -1676240
Model 2: llik = -1674557 ... best llik = -1674557
Model 3: llik = -1676240 ... best llik = -1674557
Model 4: llik = -1676240 ... best llik = -1674557
Model 5: llik = -1676240 ... best llik = -1674557
Model 6: llik = -1674557 ... best llik = -1674557
Model 7: llik = -1674545 ... best llik = -1674545
Model 8: llik = -1676240 ... best llik = -1674545
Model 9: llik = -1674557 ... best llik = -1674545
Model 10: llik = -1676240 ... best llik = -1674545
Conditional item response (column) probabilities,
 by outcome variable, for each class (row)

$rs1052133bin

	0	1
class 1:	0.5897	0.4103
class 2:	0.5813	0.4187

$rs104893751bin

	0	1
class 1:	0.9918	0.0082
class 2:	0.9922	0.0078

$rs7402844bin

	0	1
class 1:	0.0684	0.9316
class 2:	0.0797	0.9203

$rs6601606bin

	0	1
class 1:	0.9699	0.0301
class 2:	0.9594	0.0406

$rs10013040bin

	0	1
class 1:	0.5916	0.4084
class 2:	0.8073	0.1927

$rs13112390bin

	0	1
class 1:	2e-04	0.9998
class 2:	1e+00	0.0000

$rs13112358bin

	0	1
class 1:	0.0156	0.9844
class 2:	0.9993	0.0007

$rs1395479bin

	0	1
class 1:	0.5482	0.4518
class 2:	0.5854	0.4146

$rs34612342bin

	0	1
class 1:	0.9956	0.0044
class 2:	0.9961	0.0039

$rs200165598bin

	0	1
class 1:	0.9993	7e-04

class 2: 0.9995 5e-04

$rs200495564bin
0 1
class 1: 1 0
class 2: 1 0

$rs2516739bin
0 1
class 1: 0.6069 0.3931
class 2: 0.6232 0.3768

Estimated class population shares
0.9542 0.0458

Predicted class memberships (by modal posterior prob.)
0.9541 0.0459

===
Fit for 2 latent classes:
===
number of observations: 488013
number of fully observed cases: 478875
number of estimated parameters: 25
residual degrees of freedom: 4070
maximum log-likelihood: -1674545

AIC(2): 3349141
BIC(2): 3349418
G^2(2): 4977.709 (Likelihood ratio/deviance statistic)
X^2(2): 5444.928 (Chi-square goodness of fit)

[1] 3.436319
[1] 0.9975079

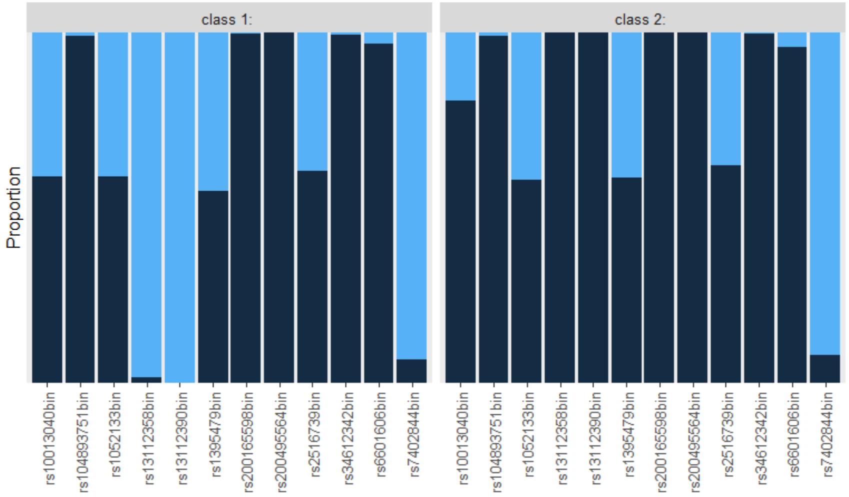

Three-class model

Conditional item response (column) probabilities,
by outcome variable, for each class (row)

```
$rs1052133bin
               0      1
class 1:  0.5776 0.4224
class 2:  0.5939 0.4061
class 3:  0.5894 0.4106

$rs104893751bin
               0      1
class 1:  0.9923 0.0077
class 2:  0.9907 0.0093
class 3:  0.9919 0.0081

$rs7402844bin
               0      1
class 1:  0.0802 0.9198
class 2:  0.0619 0.9381
class 3:  0.0691 0.9309

$rs6601606bin
               0      1
class 1:  0.9580 0.0420
class 2:  0.9709 0.0291
class 3:  0.9700 0.0300

$rs10013040bin
               0      1
class 1:  0.8078 0.1922
class 2:  0.0145 0.9855
class 3:  0.6678 0.3322

$rs13112390bin
               0      1
class 1:  0.8911 0.1089
class 2:  0.0006 0.9994
class 3:  0.0000 1.0000

$rs13112358bin
               0      1
class 1:  0.9959 0.0041
class 2:  0.0821 0.9179
class 3:  0.0000 1.0000

$rs1395479bin
               0      1
class 1:  0.5901 0.4099
class 2:  0.6118 0.3882
class 3:  0.5390 0.4610

$rs34612342bin
               0      1
class 1:  0.9962 0.0038
class 2:  0.9953 0.0047
class 3:  0.9957 0.0043

$rs200165598bin
               0     1
class 1:  0.9995 5e-04
class 2:  0.9993 7e-04
class 3:  0.9993 7e-04

$rs200495564bin
          0 1
class 1:  1 0
class 2:  1 0
class 3:  1 0

$rs2516739bin
               0      1
class 1:  0.6217 0.3783
class 2:  0.6134 0.3866
class 3:  0.6060 0.3940
```

Estimated class population shares
0.0516 0.1126 0.8358

Predicted class memberships (by modal posterior prob.)
0.0506 0.0102 0.9392

===
Fit for 3 latent classes:
===
number of observations: 488013
number of fully observed cases: 478875
number of estimated parameters: 38
residual degrees of freedom: 4057
maximum log-likelihood: -1673216

AIC(3): 3346508
BIC(3): 3346930
G^2(3): 2344.023 (Likelihood ratio/deviance statistic)
X^2(3): 2752.326 (Chi-square goodness of fit)

ALERT: iterations finished, MAXIMUM LIKELIHOOD NOT FOUND

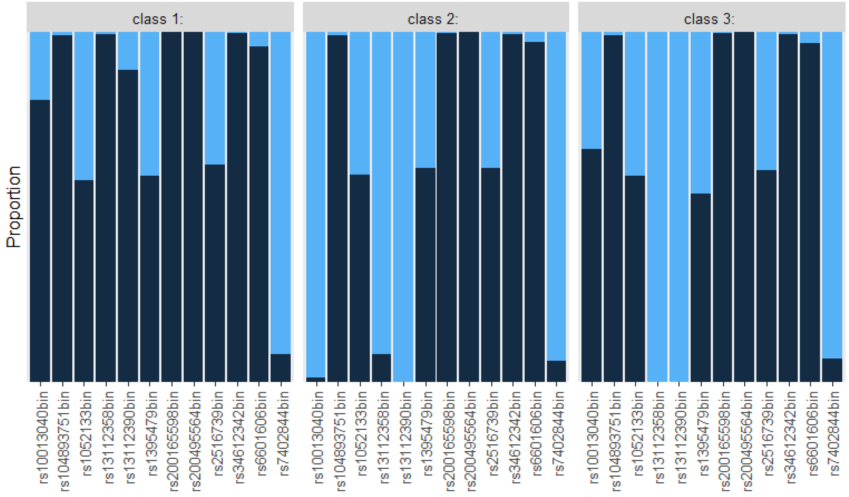

Supplement: Supplementary file 1 [file genes-15-00153-s001.zip › genes-2738464-supplementary.pdf]
